# Preventing escalation in customer service: immediate motivation and 4-week outcomes in a workplace deployment of LLM role-play training

**DOI:** 10.3389/frai.2026.1953650

**Published:** 2026-10-07

**Authors:** Kazuhiro Shidara, Sho Iwasaki, Shuji Awai, Tomoe Iida, Tatsuya Yoshimura, Takahiro Yoshioka, Kyoko Shimada, Masayuki Kiriu, Takeshi Konno

**Affiliations:** 1 Fujitsu Limited, Kanagawa, Japan; 2 Kokoro Balance Research Institute, Tokyo, Japan; 3 Department of Social Psychology, Toyo University, Tokyo, Japan

**Keywords:** customer aggression, customer service, field deployment, human–agent interaction, large language models, role-play, workplace training

## Abstract

Many customer support employees encounter aggressive customer attitudes, including overt dissatisfaction, unreasonable demands, and verbal abuse. Such attitudes do not always stem solely from customers: inadequate employee responses can amplify dissatisfaction and escalate initially manageable issues, making skills that prevent escalation critical. Recent advances in large language models (LLMs) enable role-play training with virtual interlocutors. However, role-play systems in which virtual customer attitudes shift in response to the trainee's utterances under pedagogically grounded rules remain underexplored, and evaluations of such systems rely on reaction-level measures taken immediately after use, whose relation to later workplace outcomes has not been examined in a deployment. We developed an LLM-powered training system that provides spoken dialogues with a virtual customer whose emotional expressions adapt to the employee's utterances, paired with a virtual coach for feedback. The core of the system is a state-based dialogue framework in which the virtual interlocutor's attitude escalates or de-escalates according to five binary action flags assigned to each trainee utterance, under rules co-designed with experienced call-center practitioners. We conducted a 4-week field study in an operating customer support organization, randomly assigning twelve employees to a customer-service scenario group (*n* = 6) or a structurally matched non-customer-service scenario group (*n* = 6), each completing two intervention sessions 1 week apart. The largest estimate was for motivation for behavioral change immediately after use, favoring the customer service group (*g* = 1.11, 95% CI [–0.33, 2.55]). Over 4 weeks, the between-group contrasts on change scores were small and slightly favored the comparison group (responsiveness *g* = –0.26, 95% CI [-1.55, 1.03]; productivity *g* = –0.24, 95% CI [–1.53, 1.05]); the corresponding within-group estimates in the customer service group were medium-to-large (responsiveness *g* = 0.72, 95% CI [–0.48, 1.92]; productivity *g* = 0.77, 95% CI [–0.45, 1.99]). All intervals include zero. The largest estimate fell on the measure whose content matched the customer-service material, and participants' comments followed the same pattern. We therefore argue that reaction-level measures aligned with training content cannot on their own evidence training effectiveness, and we report the framework and the deployment procedure as a basis for better-powered evaluations.

## 1 Introduction

Aggressive customer attitudes, including overt dissatisfaction, unreasonable demands, and verbal abuse toward employees, have become a pressing occupational issue that threatens employees' well-being and organizational sustainability (Dormann and Zapf, 2004; Grandey and Melloy, 2017; Jeung et al., 2018). Such behaviors—referred to as *customer harassment* in Japan and *customer aggression* in English-speaking contexts—impose substantial psychological burdens on service employees, leading to deteriorated mental health, burnout, turnover, and reputational risks for organizations (Shimada et al., 2022). From an occupational psychology perspective, related constructs such as *customer incivility, customer aggression*, and *customer mistreatment* have been linked to heightened emotional exhaustion, burnout, and turnover intentions among service workers (Grandey et al., 2007; Rupp and Spencer, 2006; Han et al., 2016). In response, the Japanese government and labor unions have issued preventive guidelines (Ministry of Health, Labor and Welfare, 2022; The Japanese Federation of Textile, Chemical, Food, Commercial, Service and General Workers' Unions, 2024). However, opportunities to acquire effective coping strategies in realistic yet psychologically safe training environments remain limited.

Dealing effectively with aggressive customers requires more than emotional endurance; it demands strategic interpersonal competence. Maintaining composure, expressing empathy, and delivering concrete solutions are known to mitigate customer frustration and prevent escalation. Prior work in service recovery shows that timely and empathic initial responses can mitigate escalation and preserve satisfaction even in emotionally charged situations (Tax et al., 1998; Van Vaerenbergh et al., 2019). When employees can manage such situations skillfully, both the perceived frequency of aggressive encounters and the associated psychological strain can decrease. Moreover, effective de-escalation contributes to smoother service recovery, reduces repeated complaints, and can enhance workplace productivity. Therefore, developing these competencies is not only a matter of individual resilience but also of organizational performance and sustainability.

Aggressive customer attitudes do not always originate solely from customers. In customer–employee interactions, inadequate or inexperienced responses can amplify dissatisfaction and escalate initially manageable issues into overtly aggressive behavior. Even when a conversation begins with a legitimate service concern, ineffective handling can shift frustration from the product or service toward the employee personally. Thus, the interaction itself—rather than the customer alone—can drive aggression. Developing customer responsiveness and communication skills is therefore critical, not only to resolve issues efficiently but also to prevent escalation and reduce psychological burden on employees (Jeung et al., 2018; Toker and Güler, 2022). Equally important is learning how to maintain composure and fall back on strategies that minimize harm when initial responses fail.

Traditional training approaches, such as case studies and in-person role-plays, provide opportunities for practice but remain limited in reproducing the intensity, variability, and unpredictability of real customer encounters (Stephens and Gwinner, 1998; Kiriu, 2021). Trainees may also feel unsafe experimenting with new strategies in conventional settings, where mistakes can be discouraging rather than instructive. From an educational design standpoint, psychologically safe environments—where participants can experiment and fail without fear of negative consequences—are known to enhance learning efficacy and reflective behavior (Edmondson, 1999; Newman et al., 2017). Role-play itself has long been an effective educational method in fields such as teacher education, nursing, and clinical training (Crow and Nelson, 2015; Brown and Chidume, 2023; Rønning and Bjørkly, 2019), and recent advances in LLMs offer a way to deliver it at scale, since LLMs can generate context-sensitive dialogue without a human partner (Shanahan et al., 2023). However, unconstrained LLM generation can produce responses that are inconsistent in tone or lack a clear pedagogical purpose, making it difficult to ensure that trainees encounter the specific interactional patterns they need to practice. This calls for a structured dialogue mechanism that ties the virtual interlocutor's behavior to explicit instructional goals while preserving conversational realism.

To address this challenge, we present an LLM-powered role-play training system for customer support employees (Figure 1). The system provides spoken dialogues with a virtual customer whose emotional expressions adapt in response to the employee's utterances, followed by reflective feedback from a virtual coach. To ensure that dialogue progression is both realistic and pedagogically consistent, the system employs a state-based dialogue framework in which customer attitudes escalate or remain manageable depending on the trainee's actions, governed by rules co-designed with experienced call-center practitioners. Our system extends prior work on conversational agents and virtual role-play into the domain of customer service by integrating adaptive dialogue control with automated feedback to support a psychologically safe training experience (Othlinghaus-Wulhorst and Hoppe, 2020; Pereira Santos et al., 2023; Louie et al., 2024).

**Figure 1:**
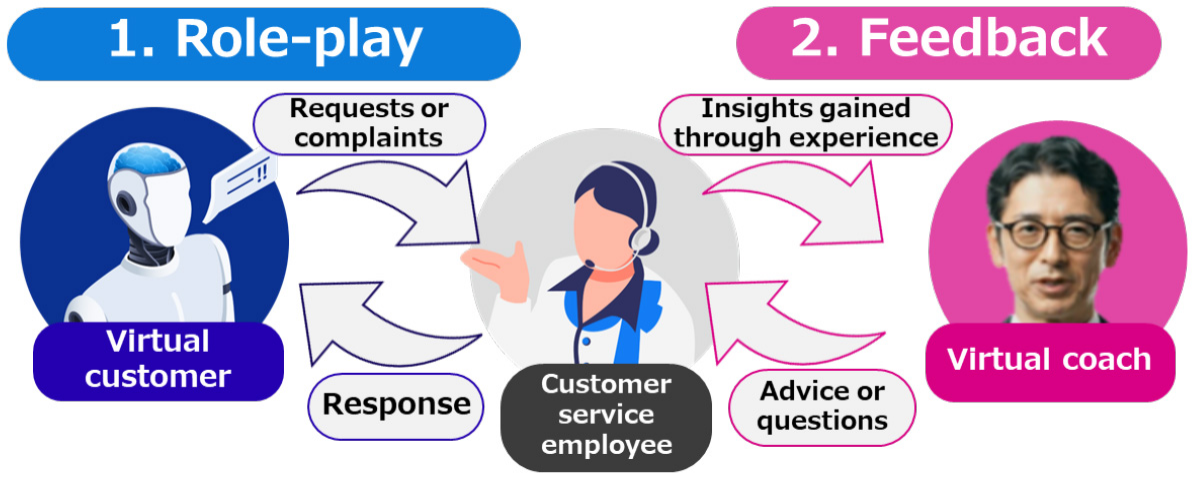
Conceptual overview of the training system. A customer support employee practices with a virtual customer agent simulating demanding interactions, followed by a feedback agent that provides personalized guidance.

Little is known, however, about how the reaction-level measures used to evaluate LLM-powered role-play relate to what happens in the workplace afterwards. Through a 4-week field deployment with customer support employees, this study examines whether the proposed system affects employees' motivation for behavioral change immediately after use, and whether customer responsiveness and workplace productivity change over the 4-week study period. To assess whether any such changes are specific to customer-service training rather than to system use in general, we compare this training against a comparison condition based on non-customer-service scenarios. We further examine, in an exploratory manner, whether the perceived frequency of challenging customer encounters changes over the study period. Because reaction-level measures taken immediately after training and self-rated outcomes measured weeks later need not move together, we also ask how these two classes of measure correspond within a single deployment. The divergence we report was observed rather than predicted, and we present it as such.

Framed in terms of behavior-change support, the system occupies a position between instruction and persuasion. It does not direct participants toward prescribed responses; instead, the virtual customer's reaction to each utterance and the virtual coach's reflective feedback let participants observe the consequences of their own choices and decide what to change. Participation was self-directed and training results did not affect workplace evaluations, so any change in how participants approached difficult customers had to arise from their own reflection rather than from external pressure. This places the system within research on human–AI interaction for voluntary behavior change, and it is the reason we treat motivation to change as an outcome in its own right rather than as a proxy for skill.

Our primary contribution is methodological: it concerns how LLM-based workplace training should be evaluated, rather than whether the system reported here improved workplace outcomes. The contributions of this study, in order of priority, are as follows:

- From a 4-week field deployment with 12 customer support employees, comparing customer-service scenarios against structurally matched non-customer-service scenarios, we report a methodological observation: the largest between-group estimate fell on a reaction-level item whose content matched one condition's material, while the 4-week outcome estimates were near zero and every interval included zero. We argue from this that reaction-level measures aligned with training content should not be treated as evidence of training effectiveness, and we report the estimates and their intervals as a basis for sample-size planning.
- We present an LLM-powered training system in which a virtual customer's emotional expressions adapt in response to the trainee's utterances, paired with a virtual coach for reflective feedback. The system was co-designed with call-center operators and supervisors, grounded in workplace practice and social psychology.
- To address the challenge of maintaining pedagogically meaningful dialogue progression, we propose a state-based dialogue framework, applicable to LLM-powered role-play training in general, that governs escalation and non-escalation of virtual interlocutor attitudes based on the trainee's classified actions, enabling realistic yet instructionally consistent interactions.

## 2 Related work

Conversational agents have increasingly been explored as tools for professional training, offering scalable and interactive alternatives to traditional case studies and face-to-face role-plays. Unlike scripted materials, these systems enable learners to engage in realistic, context-dependent dialogue while receiving immediate, personalized feedback. With recent advances in LLMs, conversational agents can now simulate complex social exchanges that capture emotional nuance, conversational flow, and situational variability more effectively than previous rule-based systems (Shanahan et al., 2023).

A growing body of research has focused on improving the controllability and pedagogical alignment of such LLM-based role-play systems. Studies on role-conditioned or structured prompting have shown how LLMs can sustain consistent personas and tone across extended conversations, improving the credibility and educational utility of simulated interactions (Louie et al., 2024; Yi et al., 2025). Other work has incorporated explicit reasoning structures—such as emotion-focused or strategy-driven prompting—to make supportive dialogue more transparent and interpretable for learners (Zhang et al., 2024). Additional approaches have emphasized dialogue-act control and theoretically grounded frameworks, such as conflict negotiation or empathy modeling, to steer interactions toward specific learning goals (Galland et al., 2024; Shaikh et al., 2024). For example, (Ramirez et al. 2023) propose a few-shot overgenerate-and-rank method that controls the generation of dialogue acts with high accuracy. From a development standpoint, phase-based design frameworks have been proposed to streamline the authoring and debugging of LLM role-play agents, contributing to more reproducible and modular training pipelines (Takagi et al., 2025; Burdisso et al., 2025). More broadly, work on controllable task-oriented dialogue (e.g., latent dialogue act models) suggests mechanisms for balancing interpretability and adaptability in response policies (Wu et al., 2023). Collectively, these studies have advanced our understanding of how to balance behavioral realism with instructional structure in AI-driven conversational learning.

Research on workplace training evaluation has long distinguished among levels of training outcome. Kirkpatrick's four-level framework separates trainees' reactions to a program, the learning it produces, the behavioral change that follows on the job, and the organizational results that may ensue (Kirkpatrick and Kirkpatrick, 2006); later work refined these categories along cognitive, skill-based, and affective dimensions (Kraiger et al., 1993). Crucially, the levels are not assumed to form a causal chain. Reviews of the framework's empirical basis have found little support for the assumption that favorable reactions lead to learning, or that learning translates into changed behavior (Alliger and Janak, 1989).

The relation between reaction-level measures and later outcomes has been examined directly. A meta-analysis of relations among training criteria found that the correlations between reactions and both learning and on-the-job behavior were generally low (Alliger et al., 1997). A later meta-analysis of the nomological network of trainee reactions found that reactions primarily capture characteristics of the training course, and that they are only modestly related to immediate knowledge outcomes and unrelated to delayed knowledge (Sitzmann et al., 2008). Whether trained behavior appears at work depends further on conditions outside the training itself: the transfer literature identifies work-environment factors, and the opportunity to perform trained tasks in particular, as conditions for transfer to be observable at all (Baldwin and Ford, 1988; Ford et al., 1992; Blume et al., 2010; Grossman and Salas, 2011). Evidence on the training methods relevant here is consistent with this picture. Simulation- and role-play-based training can produce learning gains (Salas et al., 2009), and web-based delivery is as effective as classroom instruction when the two use the same instructional methods (Sitzmann et al., 2006), but neither guarantees transfer. Within this framework, the outcomes examined in the present study span levels: the motivation item sits at the reaction level, self-rated customer responsiveness is intended as a behavioral indicator although it relies on self-report rather than observation, and self-reported productivity corresponds to the results level. These are the indicators available in an operating workplace where behavioral observation and objective performance records were not accessible to the research team; they are proxies at the behavioral and results levels rather than direct measures of them, and the strength of the evidence they can provide is bounded accordingly. The 4-week interval was chosen so that behavior and results could become observable; the reaction measure is immediate by definition.

Despite these promising developments, three critical gaps remain in the context of workplace training. First, evaluations of LLM-based training have not engaged with this measurement literature. Reaction-level measures administered immediately after use are widely reported, yet the training literature indicates that their relation to learning and transfer depends on what they ask, and that a reaction measure can separate conditions without transfer following. Whether this holds for LLM-based role-play training, and what follows for how such systems should be evaluated, has not been examined in a workplace deployment. Second, most prior studies have been conducted in short-term, laboratory, or student-based settings, leaving limited evidence regarding long-term effects on professional skills, motivation, or productivity. Indeed, comprehensive reviews of conversational agents often note the scarcity of longitudinal, real-world deployment studies as a key limitation (Bucher et al., 2024; Guan et al., 2025; Ortega-Ochoa et al., 2024). Third, while prior work has improved either the naturalness or the structural sophistication of dialogue generation, relatively few systems—especially in applied or workplace contexts—explicitly adopt phase-structured progression, such as escalation and de-escalation cycles informed by domain expertise. In reviews of pedagogical conversational agents (PCAs), scholars have noted that implementations often lack well-defined multi-phase task flows, particularly outside controlled educational settings (Ortega-Ochoa et al., 2024; Schlimbach et al., 2022; Axelsson et al., 2022).

Addressing these limitations, the present study introduces an LLM-based role-play training system for managing aggressive customer interactions. The system operates on a standard computer interface, combining controlled phase transitions and expert-guided scenario design to preserve both realism and pedagogical consistency. Through a 4-week field deployment with active customer support employees, we examine how motivation, customer responsiveness, and perceived workplace productivity changed within an actual organizational context, and how the immediate and 4-week measures correspond.

## 3 Proposed training system

As outlined in Section 1, the proposed system is designed to help customer support employees develop skills for managing aggressive customer interactions through realistic role-play and reflective feedback, while respecting participant autonomy. The system follows a two-phase workflow: (i) role-play with an LLM-driven virtual customer (Section 3.3, Figure 2), and (ii) feedback with a virtual coach (Section 3.4, Figure 3). All interactions were performed entirely through spoken utterances, with no mouse or keyboard input required except for the initial scenario selection. Before describing each phase, we first outline the scenario design principles and feedback logic that guided the system development, followed by the shared hardware and software configurations.

**Figure 2:**
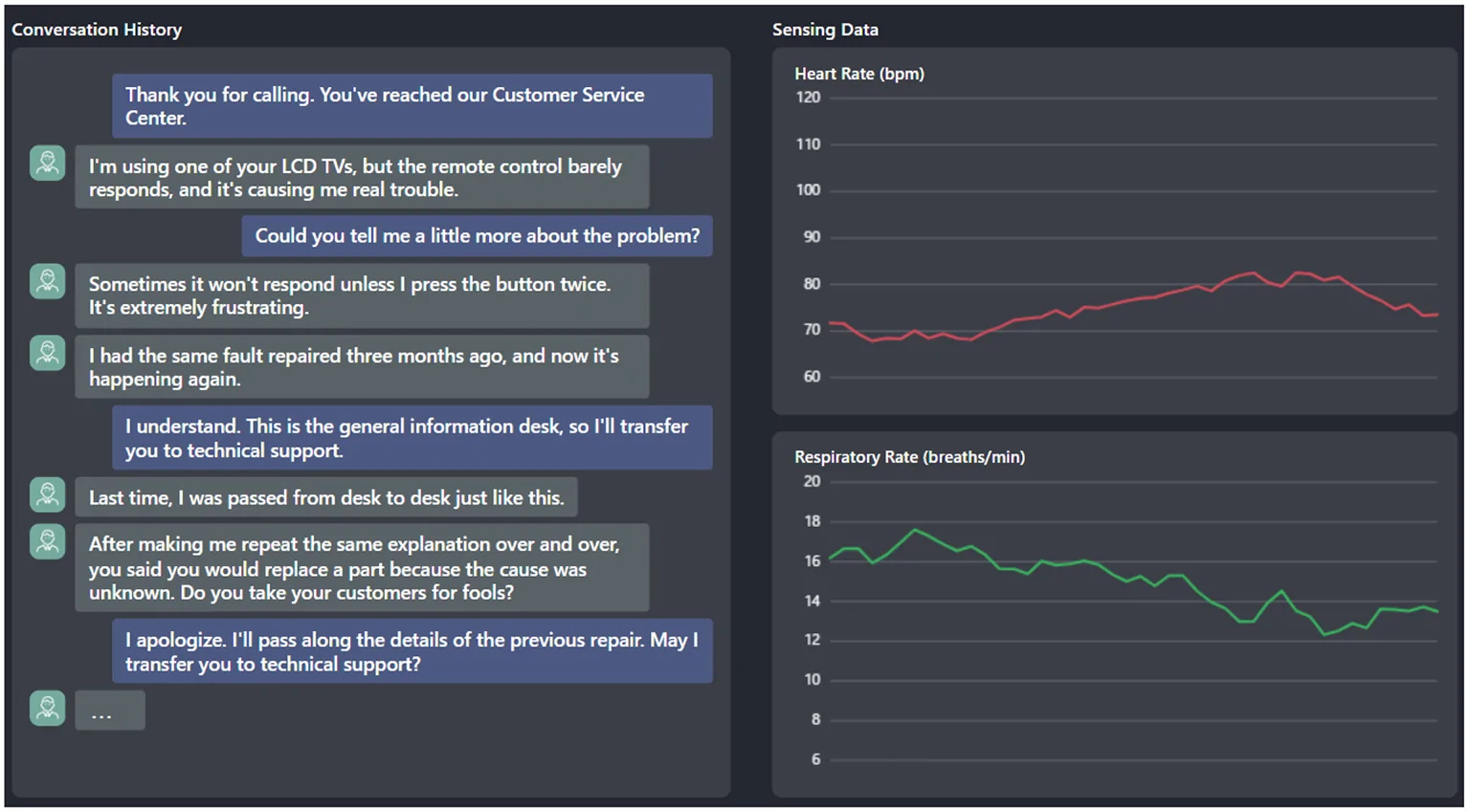
User interface of the Role-play phase. The left panel displays a real-time transcript of spoken dialogue between the user and the virtual customer, while the right panel visualizes real-time approximate heart rate (HR) and respiratory rate (RR) waveforms estimated by a 79 GHz non-contact millimeter-wave (mmWave) sensor. This display provides reference information for participants to notice changes in their physiological state during the interaction. The interface was presented in Japanese during the deployment; the labels and dialogue shown here are English renderings of the original screen. The original Japanese screenshot is provided in Supplementary material 6.

**Figure 3:**
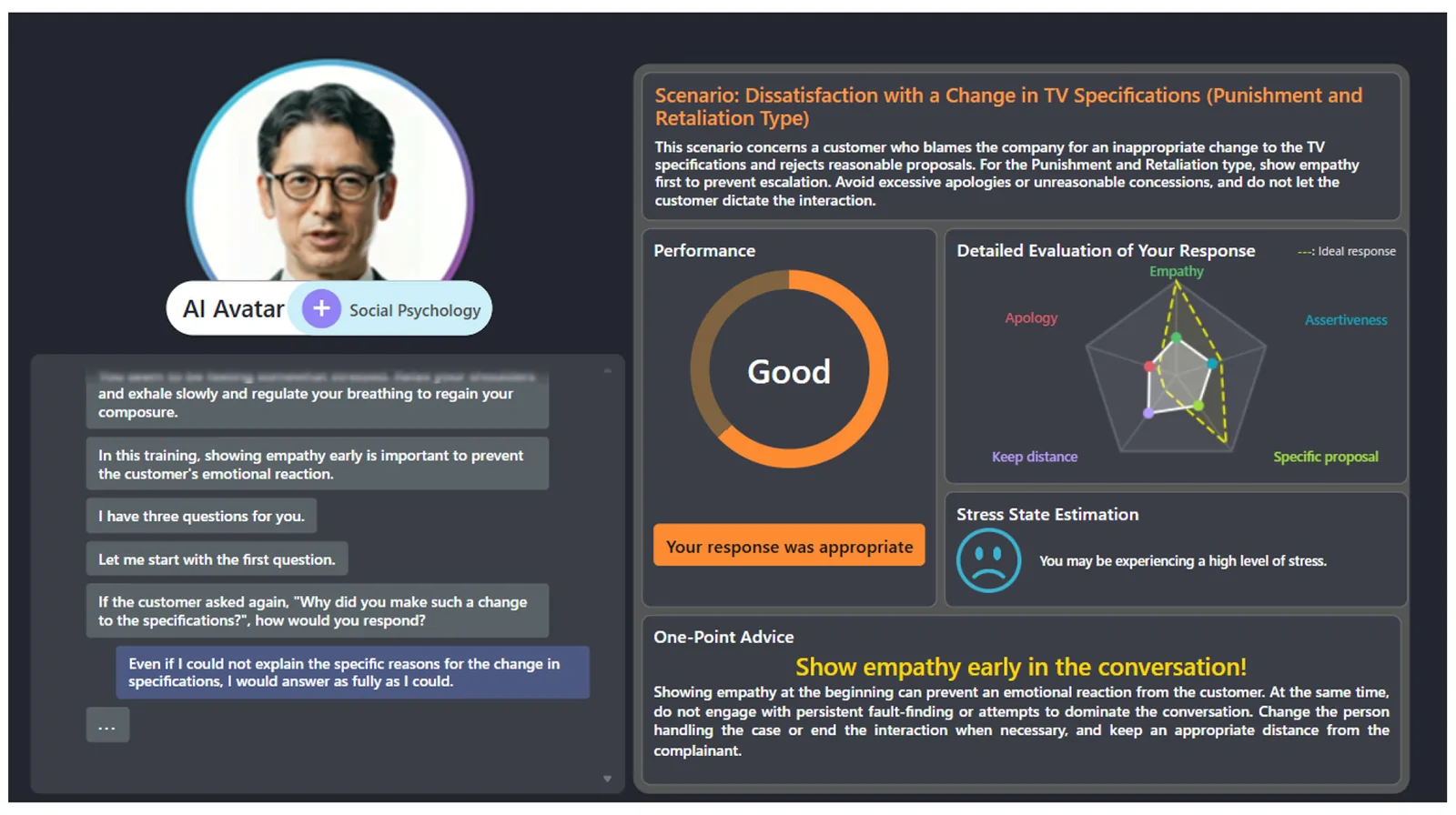
User interface of the Feedback phase. The left panel presents a reflective feedback session with a 3D virtual coach (featuring lip-sync and facial expressions), while the right panel summarizes the scenario explanation, overall performance (*Excellent, Good*, or *Needs Practice*), an action-profile radar chart for the five action dimensions, the HR-triggered reflective prompt, and advice for improvement. The interface was presented in Japanese during the deployment; the labels and dialogue shown here are English renderings of the original screen. The radar-chart labels render the on-screen labels, which correspond to the construct names used in the main text: empathy, apology, proposal, assertiveness, and distancing. The original Japanese screenshot is provided in Supplementary material 6.

### 3.1 Scenario design and feedback development

We co-designed the system with call-center operators and supervisors through six iterative design cycles. Building on a functional prototype together with workplace interviews and on-site demonstrations, we identified “secondary complaints” (escalation following inadequate initial handling) as a priority training theme. Scenarios were authored with both successful and unsuccessful trajectories and implemented within a state-based dialogue framework. Each scenario followed a three-phase structure with three exchanges per phase, where branching depended solely on the participant's classified actions (*empathy, apology, proposal, assertiveness, distancing*). These five action dimensions were defined in collaboration with call-center supervisors, drawing on occupational psychology research on service recovery and emotional labor (Tax et al., 1998; Grandey et al., 2007), to ensure both interpretability and practical relevance. This ensured that dialogue progression and affect transitions were governed by explicit instructional logic. The overall system workflow, including both the role-play and feedback phases, is illustrated in Figure 4.

**Figure 4:**
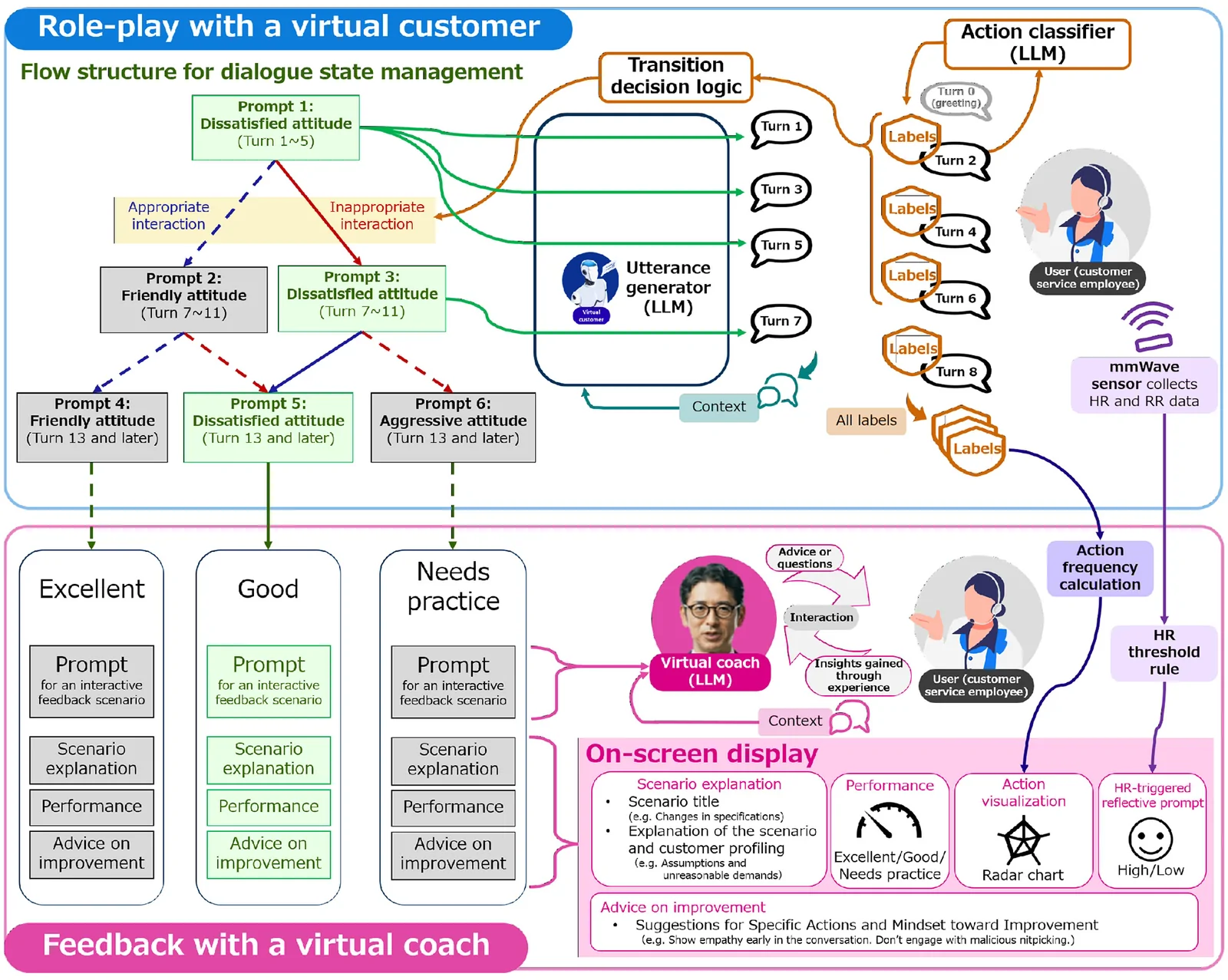
Overall system pipeline. **Top:** role-play phase. The LLM assigns five binary action flags to each user utterance—Empathy, Apology, Proposal, Assertiveness, and Distancing; the flags are independent, so a single utterance may receive several of them. The state-transition controller then determines the next dialogue state based on customer emotions (e.g., friendly → dissatisfied → aggressive). The utterance generator produces the customer's response under node-specific emotional and behavioral constraints, while the mmWave radar continuously estimates HR and RR for real-time visualization and logging. **Bottom:** feedback phase. The system aggregates action frequencies and HR/RR data and applies a rule-based threshold to generate an HR-triggered reflective prompt (presented when the threshold is exceeded), a five-dimensional radar chart visualization, and concrete advice for improvement.

#### 3.1.1 Phase logic

In Phase 1, sufficient empathy prevents escalation, while excessive distancing amplifies customer distrust. In Phase 2, combining empathy with concrete proposals or assertive clarification guides the conversation toward cooperation, whereas failure to do so results in frustration or stalemate. In Phase 3, the interaction concludes with one of three outcomes—*Excellent, Good*, or *Needs Practice*—based on cumulative action patterns, providing an interpretable summary of performance.

#### 3.1.2 Scenarios used in the study (customer service)

Two representative call-center cases were prepared under the same three-phase branching policy: *Scenario A (Repeated-Repair Complaint)*—a male customer in his 40s reports that an expensive repair did not resolve the issue (TV remote malfunctioned again); and *Scenario B (Function-Change Complaint)*—a male customer in his 70s is frustrated by usability differences in a new television model. Although the content differs, both scenarios share identical control logic and the same five-dimensional action classification, ensuring comparability.

#### 3.1.3 Outcome rubric and feedback linkage

By constraining branching to the five-dimensional classification and regulating emotional transitions, the dialogue manager reproduces realistic escalation and de-escalation at a pedagogically practical level. Outcome labels directly drive the feedback agent, which tailors reflective comments and advice to the participant's behavioral trajectory, ensuring outcome-specific and pedagogically aligned feedback.

#### 3.1.4 Assessment points and feedback refinement

Explicit assessment points (e.g., timing and quality of empathic expressions, clarity of assertive responses, excessive distancing) guided feedback design. The wording was iteratively refined to (i) surface actionable insights, (ii) align with the conversation transcript, and (iii) encourage self-reflection without inducing excessive stress.

Additionally, the same group of experienced call-center practitioners participated in the design and review of the evaluation dialogues used for system verification (Section 3.3). They confirmed the authenticity, tone, and pedagogical relevance of the reference trajectories (“Excellent”, “Good”, “Needs Practice”), with the aim of keeping the benchmark dialogues close to customer–operator interactions observed in practice.

### 3.2 Hardware and software configuration

All sessions were conducted at a standard office desk (height: 0.8 m) while participants were seated. The experimental setup consisted of a Fujitsu CELSIUS H7613 workstation running Ubuntu 22.04 LTS, equipped with an Intel Core i5-1345U CPU, an NVIDIA RTX 3500 Ada GPU (12 GB VRAM), and 32 GB of memory. A wired Jabra Evolve 20 headset (Model HSC016) was used for speech input and output, to keep audio quality consistent throughout the session.

For physiological sensing, a 79 GHz millimeter-wave multiple-input multiple-output (MIMO) radar (Calterah CAL77S244-AE) was placed on the desk approximately 0.5–1.2 m from the participant's chest, operating at a frame rate of 10 Hz. Heart rate (HR) and respiratory rate (RR) were estimated following the method described by (Li et al. 2023). The resulting approximate waveforms were displayed in real time as reference information to help participants notice changes in their physiological state during the interaction. HR and RR were components of the intervention rather than outcome measures. They were shown to participants during the interaction and, in the feedback phase, used to generate the HR-triggered reflective prompt described in Section 3.4; they were not designed or analyzed as outcome variables, and they are therefore not among the outcomes reported in this article.

The front-end, developed with Node.js and Svelte, manages WebRTC-based audio streaming, live transcription, and HR/RR waveform visualization. The Python back-end coordinates multimodal data processing, performing automatic speech recognition (Whisper; Radford et al., 2022), dialogue generation (GPT-4o; OpenAI, 2026), speech synthesis (Style-BERT-VITS2; litagin02, 2025), physiological analysis, and avatar animation using a SadTalker model (Zhang et al., 2022) for lip-synchronized output. All modules communicate asynchronously to maintain low latency and a smooth multimodal experience. All LLM-based components in both the role-play and feedback phases used GPT-4o with temperature set to 0. We note that temperature 0 reduces but does not fully guarantee output determinism, and that provider-side model updates during the study period (January–April 2025) cannot be excluded.

### 3.3 Phase 1: role-play with a virtual customer

#### 3.3.1 Front-end

In the role-play phase, users engage in spoken interaction with a virtual customer through a web interface (Figure 2). The interface displays a live transcript of both user and virtual customer utterances on the left panel, and real-time HR/RR waveforms on the right. These waveforms serve as reference information to help users notice changes in their physiological state during the conversation; in this study, they were provided identically to both groups.

#### 3.3.2 Back-end

The state-based dialogue framework introduced in Section 3.1 is implemented as a three-stage pipeline that controls the virtual customer's behavior dynamically. Through this pipeline, the virtual customer's emotional expressions adapt in response to the trainee's utterances:

1. **Action classification:** GPT-4o assigns five binary action flags to the latest user utterance—*Empathy, Apology, Proposal, Assertiveness*, and *Distancing*. The flags are independent, so a single utterance may receive zero, one, or several of them.
2. **State transition control:** A rule-based controller updates the customer's emotional state and determines the next expected reaction based on the detected action.
3. **Response generation:** GPT-4o then generates a context-constrained customer response that follows the intended emotional trajectory.

Each scenario follows the pre-defined three-phase structure introduced earlier, with multiple dialogue turns per phase. The flow branches only according to the detected actions and converges into one of three possible outcomes: *Excellent, Good*, or *Needs Practice*.

Each role-play session ends when the dialogue reaches Prompt 4, 5, or 6 in Figure 4. From that point, the virtual customer closes the conversation within three further utterances, so that a session typically spans Turns 13–17 in total. These closing utterances are generated from the preceding context, so that the session ends in a way that follows from the conversation rather than stopping abruptly.

Meanwhile, the mmWave radar continuously streams HR and RR data for synchronized visualization and logging. Detailed dialogue flows and state-transition rules are presented in Supplementary material 2. Representative example dialogues corresponding to the three outcome patterns are provided in Supplementary material 3.

#### 3.3.3 Pipeline verification

To verify the internal consistency and reliability of this three-stage pipeline, we conducted a simulation-based validation using manually defined dialogue trajectories. Each operator utterance in two customer-service scenarios—*Scenario A (Repeated-Repair Complaint)* and *Scenario B (Function-Change Complaint)*—was annotated with both the expected five-dimensional **Action classification** vector and the correct **Node transition** (e.g., “prompt-3rd-01”). The verification data and reference dialogues were developed in close collaboration with the same call-center practitioners who contributed to scenario authoring, with the aim of keeping the utterances close to the conversational tone and escalation patterns they observe in practice. During verification, each utterance was sequentially input into the system, and the dialogue controller's next-state decisions were compared with the reference trajectories. As summarized in Supplementary material 5, the system reproduced the reference trajectories on all 54 annotated utterances (nine utterances in each of six trajectories) in both *Node Transition* and *Action Classification*, in a single run at temperature 0. The reference utterances were authored for this verification and were not among the examples used to construct the few-shot classification prompt. The verification therefore speaks to the internal consistency of the controller on scripted reference trajectories, not to the validity of the action classifier on spontaneous participant speech, which was not assessed in this study.

### 3.4 Phase 2: feedback with a virtual coach

#### 3.4.1 Front-end

After each role-play session, the interface transitions to a feedback view (Figure 3). The left panel presents a 3D virtual coach (with lip-sync and facial expressions) for short reflective dialogue, while the right panel summarizes scenario explanation, performance results (*Excellent / Good / Needs Practice*), action visualization (five-dimensional radar chart representing *empathy, apology, proposal, assertiveness*, and *distancing*), an HR-triggered reflective prompt (described below), and specific advice for improvement. A representative example of the dialogue with the virtual coach is provided in Supplementary material 4.

#### 3.4.2 Back-end

The feedback engine aggregates multimodal data from the session. It computes the temporal patterns and relative frequencies of the five classified actions, then applies a heuristic threshold to the session-average HR estimated from the mmWave radar. These behavioral summaries and the resulting High/Low determination are integrated into an evaluation table that drives the feedback generation process, which includes the HR-triggered reflective prompt described below. GPT-4o produces personalized comments such as “You showed sufficient empathy but need clearer assertiveness,” or “Your responses were polite but slightly distancing,” and the SadTalker-based avatar delivers these with natural lip-synchronized animation.

The HR-triggered reflective prompt was governed by a heuristic threshold rule. We adopted 80 bpm, a value within the normal adult resting range of 60–100 bpm (American Heart Association, 2024), as the trigger; the threshold was not validated as an indicator of stress. When the session-average estimated HR exceeded this value, the virtual coach presented a supportive message such as “In such customer interactions, take a deep breath and calm yourself before continuing,” to encourage self-awareness and emotional regulation during training. This prompt is labeled “stress state estimation” on the interface to make it easier for participants to interpret. As with all AI-generated content on the feedback screen, participants were informed before the sessions that its accuracy was not guaranteed (Section 4.5). The threshold determined which participants received this prompt during the sessions.

## 4 Experimental design

### 4.1 Research hypotheses

The system described in Section 3 was designed to help customer support employees develop skills for managing aggressive customer interactions through realistic role-play and reflective feedback. However, how such a system should be evaluated when deployed in an actual workplace remains an open question. Both practitioners considering adoption of AI-based training tools and researchers seeking to understand the conditions under which LLM-powered role-play transfers to occupational outcomes need to know which measures can support which conclusions.

Based on the research aim stated in Section 1, we specified the following hypotheses in advance, together with the direction in which a training effect would be expected. As set out in Section 4.4, we evaluate them by estimation rather than by significance testing. Because the randomized allocation supports the between-group comparison, H2 and H3 are evaluated against the between-group contrast on change scores; the within-group pre–post estimates are reported descriptively. Effective customer service under pressure requires both motivation to improve and the ability to apply adaptive communication strategies. Because conventional training rarely elicits sustained motivation or realistic practice, we hypothesized that:

- **H1:** immediately after the System Use Period, participants trained on customer-service scenarios will report stronger motivation for behavioral change in customer interactions than participants trained on non-customer-service scenarios.
- **H2:** responsiveness skills for handling challenging customers will improve over the study period.

Improvements in interpersonal competence and emotional control often enhance engagement and productivity in service roles. We therefore hypothesized that:

- **H3:** self-reported productivity will improve.

In addition, if employees gain coping strategies through realistic role-play, they may perceive fewer stressful encounters. However, because the perceived frequency of challenging encounters depends largely on factors beyond the employee's control, we did not formulate this as a confirmatory hypothesis and instead examined it as an exploratory question.

### 4.2 Participants and procedure

This study was conducted from January to April 2025 in collaboration with a customer support organization in Japan. Twelve employees (10 females, 2 males; *M*_age_ = 43.0 years) met the eligibility criteria: (1) currently employed as a customer support operator and (2) able to communicate effectively in Japanese. All participants held Japanese nationality and identified as Asian. They were full-time call-center operators employed by Persol Communication Service, handling inbound customer inquiries and complaints regarding household electronic products. No monetary incentives were provided to either participants or the cooperating organization. All participants gave written informed consent and were randomly assigned (1:1) to one of two training groups. Twelve operators were available in the unit designated for participation, and the sample size was determined by this constraint rather than by a power calculation. The study was therefore designed as a feasibility deployment rather than an adequately powered effectiveness trial.

Demographic information for all participants is summarized in Table 1.

**Table 1:** Participant demographics.

Participant	Group	Age	Gender
P1	Customer service	57	Female
P2	Non-customer service	54	Female
P3	Customer service	37	Female
P4	Non-customer service	42	Female
P5	Customer service	34	Female
P6	Non-customer service	58	Male
P7	Customer service	45	Female
P8	Non-customer service	54	Male
P9	Customer service	36	Female
P10	Non-customer service	38	Female
P11	Customer service	33	Female
P12	Non-customer service	28	Female

To examine whether the proposed system specifically improves skills and motivation relevant to handling customer interactions, we compared it against a structurally matched condition involving non-customer social situations that was designed to impose comparable interactional demands. The two conditions were identical in system interface (including the real-time HR/RR reference display and all feedback components), role-play duration, spoken turn-taking, LLM-based coaching, and measurement schedule; only the *type of social situation* differed by design. Each group completed two distinct scenarios, performed sequentially on Day 1 and repeated in the same order 1 week later. Log records are incomplete for six of the forty-eight role-play sessions: two sessions were not recorded at all, and four contain no record of the feedback phase. Participants and their supervisors reported that the sessions had proceeded normally. No participant was excluded, and outcome measures were complete for all twelve participants. The overall study timeline, including the training sessions, system-use period, and follow-up evaluation, is illustrated in Figure 5.

**Figure 5:**
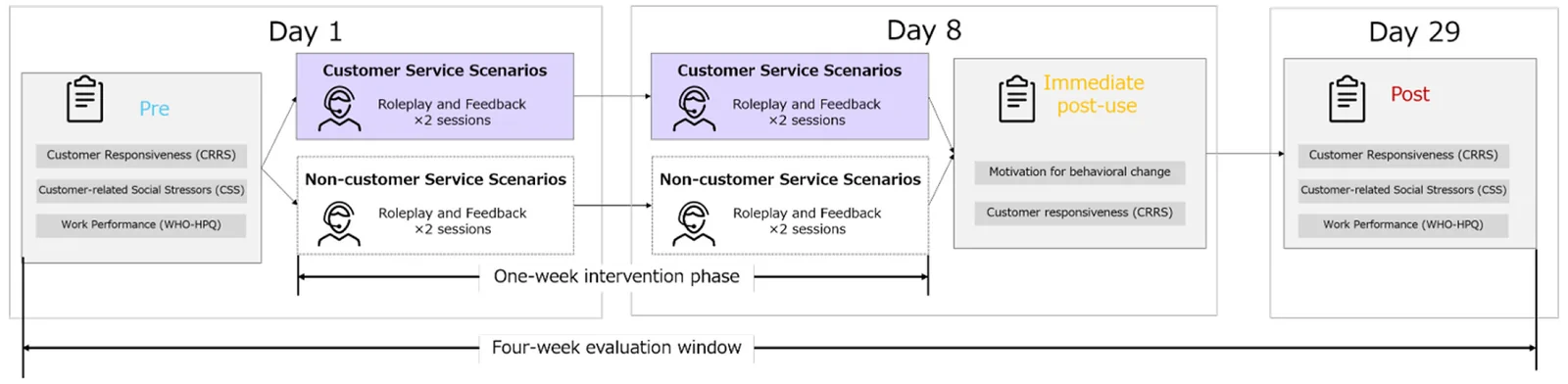
Experimental timeline. The study lasted four weeks. On Day 1, participants completed one system-use block (two consecutive role-play sessions with feedback). 1 week later (Day 8), they completed a second, identical block—together defining the *System Use Period*. Four weeks after the initial use (Day 29), participants completed a follow-up evaluation without further training, marking the end of the *4-week evaluation window*.

- **Customer service group**. Scenarios featured highly demanding or aggressive customers (Scenario A: Repeated-Repair Complaint; Scenario B: Function-Change Complaint). The virtual agent simulated emotionally charged complaints, requiring empathy expression, de-escalation, and stress management.
- **Non-customer service group**. Scenarios represented social interactions that required attentive judgment and assertive communication but were unrelated to customer service. This condition was designed to control for the general effects of using the system—such as engagement in interactive role-play and exposure to automated feedback—while excluding the customer-interaction–specific context. Specifically, this group engaged in two fraudulent-call scenarios commonly experienced in daily life: *Scenario C (Refund Scam Impersonating City Hall)*, in which a caller poses as a municipal officer offering a tax refund to elicit personal or financial information; and *Scenario D (Private “Civil Litigation Notice Center” Scam)*, in which a caller claims unpaid fees and demands immediate payment to avoid legal action. Both required information verification, deception detection, and assertive refusal, but did not involve emotional negotiation or service recovery processes. Thus, this group served as a comparison condition, intended to isolate the training effects that are specifically attributable to customer service interaction skills.

Participants were randomly assigned using simple randomization without stratification. Participants were informed that the study compared two sets of training scenarios, but were not told which set was intended as the comparison condition. Baseline questionnaires administered prior to the first system use established pre-training levels for the Customer Responsiveness Rating Scale (CRRS), the Customer-related Social Stressor Scale (CSS), and the WHO Health and Work Performance Questionnaire (WHO-HPQ), supporting within-participant change estimation and descriptive between-group comparison. Given the small sample, all outcomes are summarized as effect sizes with confidence intervals (Section 4.4).

#### 4.2.1 Questionnaire schedule

The measurement schedule was designed to align with the structure of the *System Use Period* and the *4-week evaluation window*, ensuring that each construct was assessed at theoretically appropriate intervals. These measures were grouped based on the temporal nature of their intended effects: *Immediately after use* (Day 8) for short-term and experiential outcomes, and *across the 4-week evaluation window* (Pre → Post; Day 1 to Day 29) for sustained psychological and occupational changes.

#### 4.2.2 Immediately after use (Day 8; short-term effects)

At the end of the 1-week *System Use Period*, participants completed measures capturing immediate reactions to the training. These measures corresponded primarily to H1 (motivation) and H2 (short-term responsiveness). Specifically, the following constructs were assessed:

- **Motivation for behavioral change (H1):** a single-item 7-point scale measuring participants' immediate intention to improve customer-interaction behavior after training. No pre-training measure of this item was obtained.
- **Customer responsiveness (CRRS; H2):** capturing short-term gains in self-perceived responsiveness immediately following training.

#### 4.2.3 Four-week evaluation window (Pre → Post; sustained effects)

To examine whether the training effects were retained over the 4-week window, the following instruments were administered both at baseline (Pre; Day 1) and follow-up (Post; Day 29). Because the CSS and WHO-HPQ assess ongoing experiences in daily work and life, a 4-week interval was chosen to capture meaningful changes beyond short-term fluctuations.

- **Customer responsiveness (CRRS; H2):** measuring sustained improvement in customer-handling efficacy.
- **Work performance (WHO-HPQ; H3):** assessing self-reported productivity and workplace functioning.
- **Customer-related social stressors (CSS; exploratory):** although not included as a formal hypothesis—because the perceived frequency of challenging encounters depends largely on factors beyond the employee's control—we assessed CSS to explore whether the training was accompanied by changes in perceived exposure to difficult customers.

### 4.3 Outcome measures and evaluation metrics

To characterize what the deployment produced, we employed multiple quantitative and qualitative measures capturing motivational, behavioral, and occupational outcomes. Each measure corresponded to one or more hypotheses: motivation for behavioral change (H1), customer responsiveness (H2), and workplace productivity (H3), along with an exploratory assessment of perceived customer-related stressors.

#### 4.3.1 Customer responsiveness

Participants' perceived self-efficacy and ability to handle difficult customers were assessed using the newly developed CRRS. The CRRS consists of 30 items rated on a 5-point Likert scale (total scores range from 30 to 150), with higher scores indicating stronger skills in managing challenging customers. The development process and finalized items are presented in Supplementary material 1. The scale was developed by the authors. In the development sample of 1,299 service-sector workers, internal consistency for the 30-item total score was high (Cronbach's α = 0.94) and the standard error of measurement was 4.05 points. Its test–retest reliability has not been established; Section 6.4 discusses what this implies for interpreting the observed change scores.

#### 4.3.2 Customer-related social stressors

Measured using the Japanese version of the CSS (Dormann and Zapf, 2004; Shimada et al., 2023). This 15-item scale assesses the frequency of difficult customer interactions (1 = never deal with, 5 = always deal with), with higher scores reflecting greater daily exposure to customer-related stressors.

#### 4.3.3 Motivation for behavioral change

Measured using a single-item 7-point Likert scale (1 = no intention at all, 7 = absolutely will change). This item captures motivation for behavioral change following training sessions.

#### 4.3.4 Subjective productivity

Evaluated using a single item from the WHO-HPQ (Kessler et al., 2003; Suzuki et al., 2014). This 11-point scale (0 = lowest, 10 = highest) measures subjective productivity, with higher scores indicating better self-perceived work performance.

#### 4.3.5 Open-ended responses

In addition to the quantitative measures, participants provided short open-ended comments after each measurement point, describing what they had attended to in customer interactions since the previous measurement and any difficulties they had encountered. Two authors independently read all responses and grouped them by content in relation to the outcomes above, with disagreements resolved by discussion; representative excerpts are quoted with participant IDs (e.g., P3, P7). Given the small number of participants, we describe which themes appeared in each condition but do not quantify their relative frequency or treat any difference in frequency as an estimate of a population difference.

### 4.4 Data analysis

Given the small sample (*n* = 6 per group), we adopted an estimation approach rather than null-hypothesis significance testing. For each outcome we report the effect size as Hedges' *g* (small-sample corrected) with its 95% confidence interval (CI), and we interpret the direction and precision of these estimates. Accordingly, we do not report *p*-values and do not apply a multiplicity correction.

Motivation was evaluated as a between-group contrast at Day 8 (Customer service minus Non-customer service), with *g* computed for independent samples. Customer responsiveness and productivity were evaluated in two complementary ways: as within-participant pre–post contrasts (Day 1 → Day 29) within each group, with paired *g*; and as a between-group contrast on change scores (Customer service minus Non-customer service), with *g* computed for independent samples. We report both because the two quantities answer different questions: the within-group estimate describes change over time in each group, whereas the change-score contrast is the comparison that the randomized allocation supports. The exploratory CSS measure is reported in the same two ways. For customer responsiveness we additionally report the within-group pre–post contrast at Day 8, immediately after the System Use Period, since the scale was administered at that point as well.

Confidence intervals for paired effect sizes were computed as

$$g\pm t_{.975,n-1}\sqrt{\frac{1}{n}+\frac{g^{2}}{2\left(n-1\right)}},$$

and the interval for the independent-samples contrast as

$$g\pm t_{.975,\nu }\sqrt{\frac{n_{1}+n_{2}}{n_{1}n_{2}}+\frac{g^{2}}{2\left(n_{1}+n_{2}\right)}}.$$

For the Day 8 motivation contrast, ν was obtained from the Welch–Satterthwaite approximation; for the between-group contrasts on change scores, ν = *n*_1_+*n*_2_−2. All estimates were computed in Python 3.10 with SciPy 1.15. We describe these choices in words as well, since the formulas alone do not convey what the estimates can support. Cohen's *d* is upwardly biased when samples are small, and the bias is not negligible at six participants per group; Hedges' *g* multiplies *d* by a correction factor slightly below one that approaches one as the sample grows, so the values reported here are smaller in magnitude than the uncorrected ones would be. Two forms of *g* appear. The paired form standardizes a pre–post difference by the standard deviation of the change scores and is used for the within-group estimates; the independent-samples form standardizes a difference between two groups by their pooled standard deviation and is used for the Day 8 motivation contrast and for the between-group contrasts on change scores. The two are not on a common scale, and we do not compare their magnitudes. Each confidence interval was formed by adding and subtracting a multiple of the estimate's standard error, with the multiplier taken from the *t* distribution at the corresponding degrees of freedom; the interval widens as the sample shrinks, which is why the intervals reported here are wide. An interval that includes zero means that the data are compatible with a range of values that includes no difference. It does not mean that the difference is zero, and it does not establish that the training had no effect: with six participants per group, intervals of this width are expected whether or not a difference exists. Quantitative estimates were interpreted alongside participants' open-ended responses.

### 4.5 Ethical considerations

This study was reviewed and approved by the Research Ethics Committee of the Graduate School of Sociology, Toyo University (Approval ID: P240044). Participants provided written informed consent, and all sessions were conducted during regular work hours. All collected data were anonymized, and only the customer support manager retained the key linking participant identities to research IDs; the research team had no access to participants' names or contact details. Participants were explicitly informed of their right to withdraw at any time and to request deletion of their data. Participants were also informed before the sessions that all AI-generated content on the feedback screen, including the performance labels and the HR-triggered reflective prompt, was produced automatically and that its accuracy was not guaranteed. The model version and decoding settings are reported in Section 3.2, and the risk of evaluative misuse is discussed in Section 6.4.

Because the system utilized third-party APIs for LLM-based response generation, participants were clearly informed during consent that their speech transcripts would be transmitted to an external provider for temporary processing. Importantly, no real customer data were used; all dialogue scenarios and response contents were artificially generated solely for research purposes. To minimize risks of personal information leakage, the scenarios were designed not to include prompts requesting or revealing employees' personally identifiable information (e.g., names, contact details). Participants were also instructed in advance not to disclose any personal or company-related information during sessions. As a result, no transmitted data contained real customer information or employee identifiers.

Training sessions were conducted in private rooms with only participants and company supervisors present for safety and procedural oversight, thereby reducing the risk that surrounding workplace conversations or sounds were unintentionally captured. All transcripts were anonymized before analysis, with any identifiers or sensitive content removed, thereby minimizing the risk of re-identification. We note that, because the cooperating organization is named and Table 1 reports participant-level characteristics, participants may remain identifiable to colleagues within that organization.

## 5 Results

Analyses followed the plan in Section 4.4: a between-group contrast at Day 8 for motivation, and, for responsiveness and productivity, both within-group pre–post contrasts and a between-group contrast on change scores; the exploratory CSS measure is reported in the same two ways. Throughout we report **Hedges'** **g** with **95% CIs**. Where informative, quantitative outcomes are interpreted together with qualitative evidence.

### 5.1 H1: Motivation for behavioral change

Figure 6 shows that motivation for behavioral change on Day 8 was higher in the Customer service group than in the Non-customer service group, with a large effect size whose 95% confidence interval included zero (Hedges' *g* = 1.11, 95% CI [−0.33, 2.55]). The mean difference (Customer − Non-customer) was 1.33, 95% CI [−0.15, 2.82]. The point estimate suggests a practically meaningful difference in short-term motivation between the two scenario conditions, but the wide interval—expected given the small sample—means its magnitude should be read as provisional. Because motivation was not measured at baseline, this contrast does not establish an increase over time within either group.

**Figure 6:**
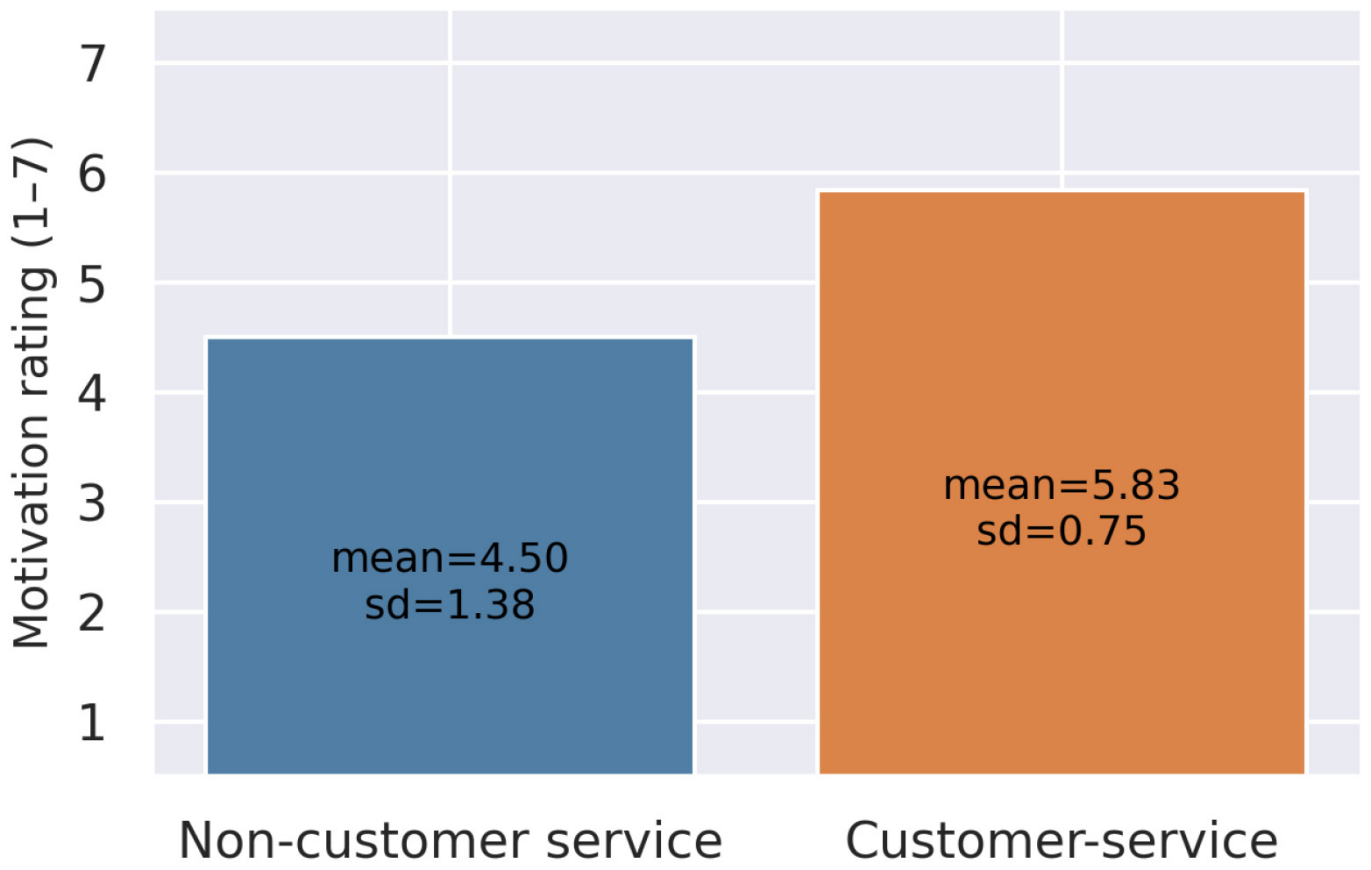
Inter-group comparison of motivation for behavioral change on Day 8. Bars indicate group means ± SD.

The open-ended responses were consistent with this pattern: participants in the Customer service group reported stronger motivation to improve interpersonal strategies, such as “I want to use more expressions that empathize with customers' emotions” (P1), “I want to take a breath and sense the situation before responding” (P3), and “I want to increase words that convey empathy” (P9). Alongside this motivation, participants pointed to obstacles: P9, who wanted to convey more empathy, also had “no chance to apply the training,” and P7 found that “it was difficult to know how to respond properly.” In contrast, participants in the Non-customer service group (e.g., P4, P8, P10) often noted that the training context felt less relevant to their work roles.

### 5.2 H2: customer responsiveness (CRRS)

#### 5.2.1 Evaluation immediately after the system use period

Immediately after the 1-week System Use Period, both groups showed negligible-to-small pre–post effects. In the Customer service group, the CRRS score was 95.67 (*SD* = 7.92) before training and 93.83 (*SD* = 11.82) after training (Hedges' *g* = −0.22, 95% CI [−1.29, 0.84]). In the Non-customer service group, scores were 108.00 (*SD* = 8.27) before training and 109.67 (*SD* = 9.73) after training (*g* = 0.20, 95% CI [−0.86, 1.26]).

#### 5.2.2 Evaluation in the 4-week window

At the 4-week follow-up, the between-group contrast on change scores was small and slightly favored the comparison group (*g* = −0.26, 95% CI [−1.55, 1.03]), reflecting a mean gain of +2.83 points in the Customer service group and +4.33 points in the Non-customer service group. H2 was therefore not supported. The corresponding within-group estimates are shown in Figure 7: a medium-to-large pre–post effect in the Customer service group, with a 95% confidence interval that included zero (Hedges' *g = 0.72*, with spaces around the equals sign, as elsewhere in the article. 95% CI [−0.48, 1.92]), and a smaller effect of the same direction in the Non-customer service group (*g* = 0.54, 95% CI [−0.60, 1.68]). Individual pre–post trajectories are depicted in Figure 8. Five of six participants in the Customer service group showed improvement (P1, P3, P5, P9, P11), whereas four of six improved in the Non-customer service group (P4, P6, P8, P10).

**Figure 7:**
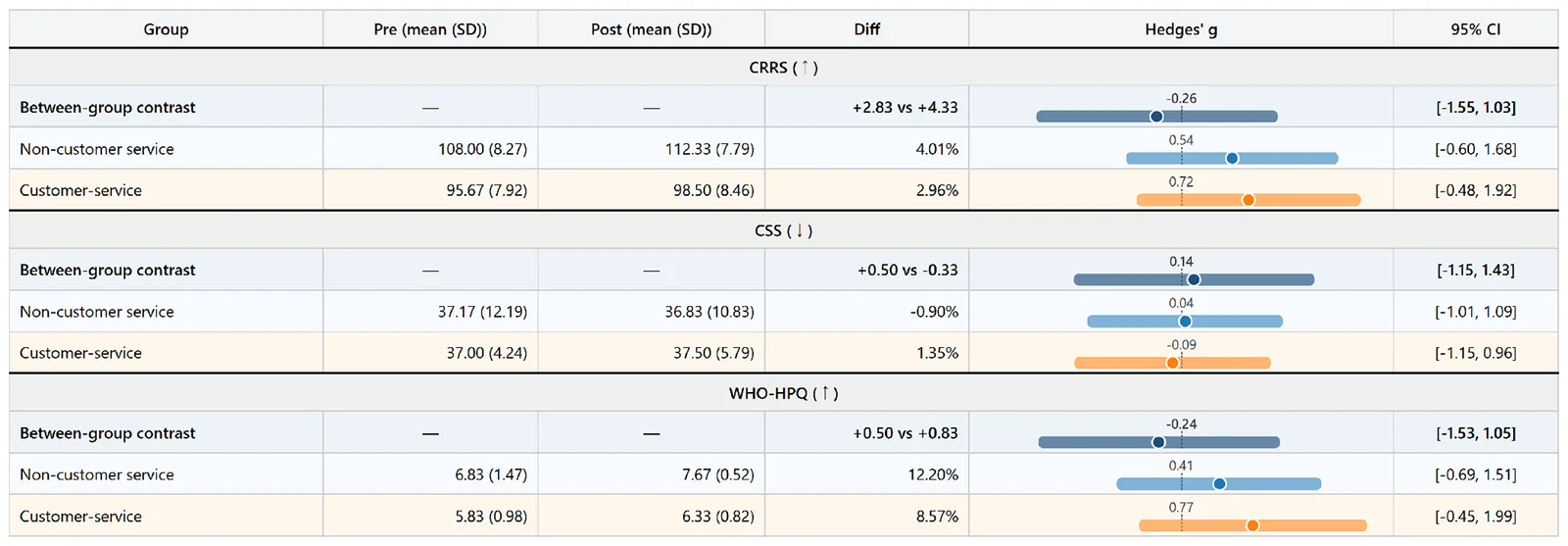
Between-group change-score contrasts and descriptive within-group pre–post estimates at the 4-week evaluation window for three outcome measures: CRRS, CSS, and WHO-HPQ. For each measure, the first row reports the between-group contrast on change scores, which is the comparison supported by the randomized allocation, and gives the mean change in each group (Customer service vs. Non-customer service). The two rows that follow report the descriptive within-group estimates for each group (*n* = 6 each): means (SD) at Pre and Post, relative percent change (*Diff* = (Post–Pre)/Pre), Hedges' *g*, and 95% confidence intervals (CI). In the effect-size column, the bar spans the 95% confidence interval, the marker indicates the point estimate, and the dashed line marks zero. All confidence intervals include zero. Arrows denote the direction interpreted as improvement (↑: higher is better; ↓: lower is better). For the within-group CSS estimates (↓), Hedges' *g* is signed so that positive values indicate improvement (i.e., a decrease in scores); the between-group CSS contrast is computed on raw change scores and is therefore signed in the opposite direction (Section 5.5).

**Figure 8:**
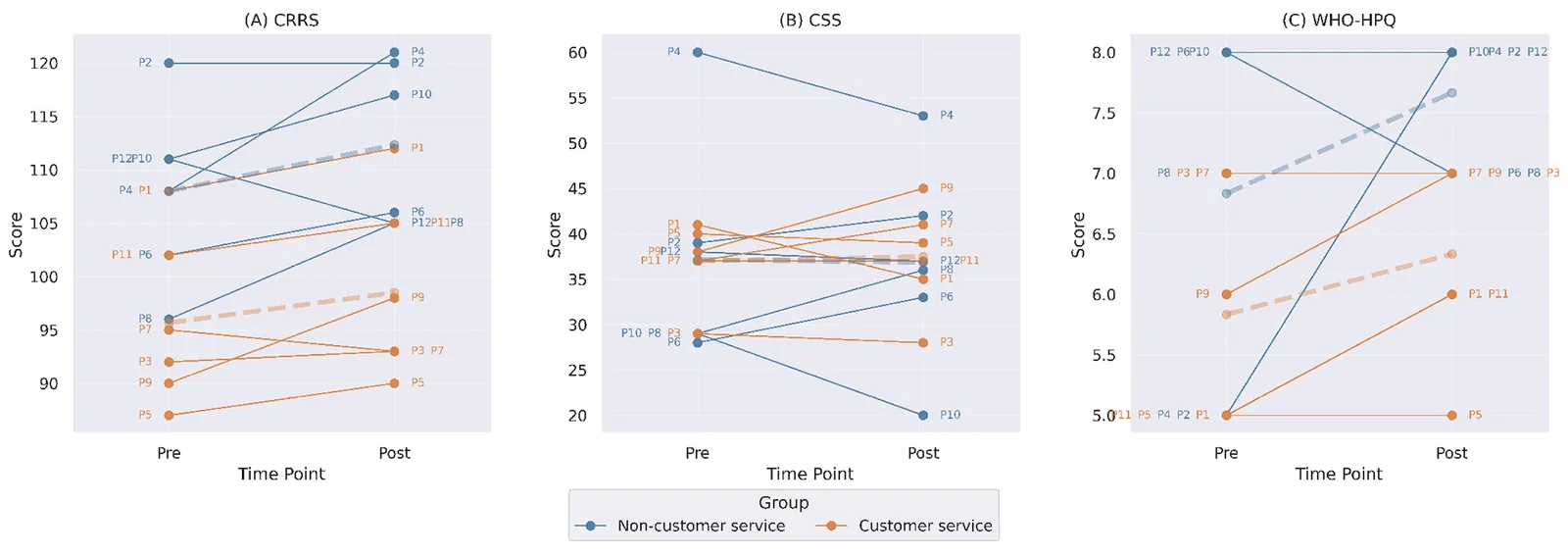
Spaghetti plots of individual pre–post score changes at the 4-week evaluation window. The horizontal axis represents the measurement time points (Pre, Post), and the vertical axis indicates scale scores. Blue lines correspond to the *Non-customer service* group and orange lines to the *Customer service* group. Three outcome measures are shown: **(A)** CRRS, **(B)** CSS, and **(C)** WHO-HPQ. Thin solid lines represent individual trajectories, while faint dashed lines depict group means.

Participants in the Customer service group described shifts toward calmer and more empathetic communication. For instance, P1 emphasized “valuing empathy and consideration,” P3 referred to “remaining calm in conversation,” and P7 reported “preparing empathetic words.” Others highlighted context-sensitive responses such as “being able to express appropriate thanks depending on the situation” (P1) and “accepting as much as possible” (P11). In contrast, some noted barriers, with P5 reporting that “responses did not proceed as expected.” Participants in the Non-customer service group mentioned “maintaining a hearing attitude” (P10), “keeping distance with the customer” (P12), and “the training was not useful for my actual work” (P6). Together, these accounts describe self-reported shifts in response strategies, alongside marked heterogeneity in how applicable participants found the training.

### 5.3 H3: workplace productivity (WHO-HPQ)

The between-group contrast on change scores was likewise small and slightly favored the comparison group (*g* = −0.24, 95% CI [−1.53, 1.05]; mean gains of +0.50 and +0.83 points, respectively). H3 was therefore not supported. The corresponding within-group estimates are shown in Figure 7: a medium-to-large pre–post effect on productivity in the Customer service group, again with a 95% confidence interval that included zero (Hedges' *g* = 0.77, 95% CI [−0.45, 1.99]), and a smaller effect in the Non-customer service group (*g* = 0.41, 95% CI [−0.69, 1.51]). Individual patterns illustrated in Figure 8 show that three of six participants in the Customer service group (P1, P9, P11) improved, and two of six in the Non-customer service group (P2, P4).

The open-ended responses were consistent with these patterns. Participants in the Customer service group described behavioral adjustments such as “considering words of empathy” (P7), “accepting as much as possible” (P11), and “expressing appropriate thanks depending on the situation” (P1). Others noted limited realism or opportunity for application (“no chance to apply the training,” P9; “responses did not proceed as expected,” P5). In the Non-customer service group, some mentioned “being able to respond more calmly” (P10) or “keeping composure under pressure” (P12), while others emphasized “lack of applicability” (P6) or “insufficient realism” (P8).

### 5.4 Divergence between the immediate motivation item and the 4-week outcome scores

The estimates did not all point in the same direction. For the immediate motivation item, the point estimate favored the Customer service group by a large margin (Section 5.1). For the 4-week outcome scores, the two groups improved to a similar degree, and the comparison group's mean gains were numerically larger for both measures. The larger standardized estimates in the Customer service group therefore reflect a smaller variance of change scores rather than larger average gains. Consistent with this, the between-group contrasts on change scores were small and, for both outcomes, slightly favored the comparison group (Sections 5.2 and 5.3). We therefore do not read the within-group estimates as evidence that the gains were specific to customer-service scenario content. Participants' comments were consistent with this divergence: those in the Customer service group referred to their own complaint handling, whereas those in the comparison group referred to the training as unrelated to their work (“the training was not useful for my actual work,” P6). Because the motivation item asks specifically about customer interactions, whereas the comparison condition trained fraudulent-call handling, its content matches one condition's material. A measure of this kind can produce a large between-group estimate whether or not the training transferred, and we therefore treat the estimate observed here as uninformative about effectiveness.

### 5.5 Exploratory analysis: perceived frequency of challenging customers (CSS)

Although not included as a formal hypothesis, we examined whether the perceived frequency of challenging customer encounters changed over the study period. The between-group contrast on change scores was also negligible (*g* = +0.14, 95% CI [−1.15, 1.43], computed on raw change scores, so that positive values indicate a larger increase in CSS—that is, less improvement—in the Customer service group). As lower CSS indicates fewer stressful encounters, Figure 7 shows correspondingly negligible group-level effects (Customer service: *g* = −0.09, 95% CI [−1.15, 0.96]; Non-customer service: *g* = 0.04, 95% CI [−1.01, 1.09]). For the within-group CSS estimates, Hedges' *g* is signed so that positive values indicate improvement (i.e., a decrease in scores). Both groups' mean scores were around 35–38 points on a scale ranging from 15 to 75, corresponding to occasional rather than frequent exposure to difficult or aggressive customers. Individual-level variations are shown in Figure 8, where improvements were observed for three participants in each group (Customer service: P1, P3, P5; Non-customer service: P4, P10, P12). Seven participants reported no encounters with challenging customers during the study period (P1, P3, P5, P9, P10, P11, P12); two described such encounters (P4, P7), and the remaining three (P2, P6, P8) did not comment on this point. Among those who did, qualitative data suggested behavioral adaptation rather than frequency change—for example, “thinking about words of empathy” (P7) and “trying to respond in a way that empathizes with the customer's situation” (P4).

## 6 Discussion

### 6.1 Main findings

This study deployed an LLM-powered role-play training system in an operating customer support organization and examined how motivation, self-rated responsiveness, and self-reported productivity changed. The central observation is that the largest between-group estimate fell on the immediate motivation item, whose content matches the customer-service material; the 4-week outcome scores showed estimates near zero, slightly favoring the comparison group. All of these intervals include zero, including the interval for the motivation estimate. What differs across the measures is therefore the size and direction of the point estimates rather than the strength of the evidence. The outcomes reported here concern motivation, responsiveness, and productivity; psychological safety was addressed through the procedural safeguards described in Section 6.3 and is not among the outcomes evaluated in this article.

#### 6.1.1 H1 (Motivation for behavioral change)

The largest between-group estimate was for short-term motivation for behavioral change, favoring the Customer service group, with an interval that included zero (*g* = 1.11, 95% CI [−0.33, 2.55]), alongside participants' self-reflections emphasizing empathy and calm communication (e.g., P1: “valuing empathy and consideration”; P3: “remaining calm in conversation”; P9: “increasing words that convey empathy”).

#### 6.1.2 H2 (Customer responsiveness)

The comparison that the randomized allocation supports is the between-group contrast on change scores, which was *g* = −0.26 (95% CI [−1.55, 1.03]), slightly favoring the comparison group. The quantitative outcomes therefore provide no support for an alignment effect. The within-group estimates point the other way: responsiveness skills (CRRS) showed a medium-to-large pre–post effect in the Customer service group (*g* = 0.72, 95% CI [−0.48, 1.92]) and a smaller effect of the same direction in the Non-customer service group (*g* = 0.54, 95% CI [−0.60, 1.68]). At the individual level, most participants in the Customer service group (P1, P3, P5, P9, P11) improved, consistent with self-reported gains in regulating tone, responding empathetically, and managing situational stress. As set out in Section 5.4, these within-group patterns reflect a smaller variance of change scores rather than larger average gains, and they are not the comparison the design supports. The difference participants themselves described was in how applicable they found the training, which the open-ended responses report rather than the quantitative estimates.

#### 6.1.3 H3 (Workplace productivity)

The between-group contrast on change scores for perceived productivity (WHO-HPQ) was *g* = −0.24 (95% CI [−1.53, 1.05]), again slightly favoring the comparison group, while the within-group estimate in the Customer service group was medium-to-large (*g* = 0.77, 95% CI [−0.45, 1.99]). Neither H2 nor H3 was supported.

#### 6.1.4 Exploratory: perceived frequency of challenging encounters

The perceived frequency of challenging encounters (CSS) showed negligible effects. Nonetheless, the open-ended responses pointed to subtle behavioral adaptation—participants mentioned “thinking about words of empathy” (P7) and “trying to respond in a way that empathizes with the customer's situation” (P4)—suggesting reframing of stressful interactions rather than a change in their frequency.

### 6.2 Interpretation and design implications

Taken together, the findings suggest that the proposed system may function primarily as a **motivational and reflective catalyst**. Heightened motivation to change may precede gradual behavioral change, a sequence consistent with experiential learning theories (Kolb, 2014; Schön, 2017). Participants initially recognized the difficulty of handling aggression (“responses did not proceed as expected,” P5) but later described calmer and more empathic strategies (“remaining calm,” P3).

The Non-customer service group showed CRRS gains of comparable magnitude, and in raw terms slightly larger ones. One reading is that exposure to controlled, high-tension dialogue prompts reflective recalibration of one's communication patterns regardless of job role, so that emotional regulation skills generalize beyond customer-facing contexts. This reading is consistent with prior work showing that simulation-based and emotionally demanding practice environments, followed by structured reflection, foster durable behavioral adaptation (Fanning and Gaba, 2007; Rudolph et al., 2008), and would suggest that domain-general role-play can complement specialized training when designed with empathy-oriented framing. A second reading, which the present design cannot rule out, is regression toward the mean from the comparison group's higher baseline (Section 6.4).

From a design perspective, these results advocate for **continuous and adaptive training**. If motivational activation precedes behavioral improvement, periodic re-engagement cycles could sustain learning and prevent decay. The HR and RR waveforms shown during training may also serve, in future systems, as a real-time signal for adjusting scenario intensity or feedback timing, in line with research on affect-sensitive and adaptive learning environments (Picard, 1997; D'Mello and Graesser, 2012; Kapoor et al., 2007); we detail this direction in Section 6.5. A further account for the absence of divergence in the 4-week outcome scores is limited opportunity for application. Most participants reported no encounters with challenging customers during the study period (Section 5.5), and several described having had “no chance to apply the training” (P9, P7). Where the trained behaviors cannot be enacted, transfer cannot be observed regardless of whether it occurred. Deployments of this kind should therefore measure or verify exposure to the target situation rather than assume it.

Independently of these outcome estimates, the deployment shows what the state-based framework contributes as a design. Separating action classification, state transition, and constrained utterance generation makes each layer inspectable and revisable in isolation, which is not the case when a single prompt governs the whole exchange. The transition rules encode pedagogical priorities rather than conversational plausibility, and this is what allowed practitioners to author and revise them. We expect the separation of layers and the rule-based transition logic to transfer to other interpersonal training domains, whereas the five action dimensions and the phase priorities are specific to service recovery and would need to be redefined with domain experts.

### 6.3 Safe and responsible innovation statement

The system was developed and deployed under strict ethical and safety considerations. Virtual customer agents were carefully designed to avoid biased or discriminatory expressions. Participants were informed that training results would not affect workplace evaluations. A supervisor was present during all sessions, and the research team monitored system responses in real time so that a session could be stopped if a participant appeared distressed or the system produced inappropriate content. Neither occurred during the study. These procedures were intended to protect participants from foreseeable harm and illustrate a responsible application of LLMs in sensitive workplace contexts. We report them as safeguards; this article does not evaluate psychological safety as an outcome.

### 6.4 Limitations

This article makes two claims: that the state-based dialogue framework can be implemented and operated in a real workplace, and that a 4-week deployment produced the estimates reported above. It does not claim that the training is effective, that the LLM-generated feedback is valid as an assessment, or that the physiological display improves outcomes. Each of those would require a study designed for that purpose, with a correspondingly different sample and set of measures. The limitations below are stated within this scope.

Several limitations should be acknowledged. First, the sample size was small (*n* = 6 per group) and drawn from a single organization, which limits both statistical power and generalizability. A confirmatory study would require a substantially larger sample, preferably drawn from more than one organization, with the sample size set by a formal power calculation and with randomization stratified or blocked on baseline responsiveness. We therefore specified the outcomes and the direction in which a training effect would be expected before data collection, and we base interpretation on effect sizes and confidence intervals. The confidence intervals for the primary effects include zero, and the findings should therefore be read as indicative rather than confirmatory. Second, all outcomes relied on self-reported measures. Behavioral indices such as coded call recordings would have required access to live customer interactions, which the data-collection agreement with the cooperating organization did not permit. In particular, motivation for behavioral change (H1) was assessed with a single item administered only after training, so pre-existing group differences in motivation cannot be excluded. As set out in Section 5.4, the item's content also matches one condition's material, and we therefore read this contrast as a domain-aligned motivational response rather than a general motivational advantage. Third, scenario difficulty and structure were fixed rather than adaptive, which may have influenced engagement or learning progression. Fourth, the 4-week outcomes presuppose that participants encountered the situations the training addressed, and this pre-supposition was not met for most of the sample: seven of the twelve participants reported no encounters with challenging customers during the study period (Section 5.5). For those participants the outcome scores cannot distinguish an absence of training effect from an absence of opportunity to exhibit one. We did not measure or verify exposure to the target situation.

The system also displayed real-time HR/RR waveforms and, where the threshold was exceeded, an HR-triggered reflective prompt; both were provided identically to both groups and were not evaluated as an intervention in this study. Because they were common to both conditions, they do not account for the observed between-group differences. Their effectiveness, and the validity of the underlying estimation, are outside the scope of this article.

The largest between-group estimates were for short-term motivation (H1) and for perceived applicability rather than for the quantitative pre–post gains; these should be interpreted with caution, as domain alignment rather than the training method *per se* may have moderated them. Participants in the Non-customer service group explicitly noted the limited relevance of the training scenarios to their daily work (e.g., “the training was not useful for my actual work,” P6), suggesting that the specificity of scenario content to one's professional context may be a necessary condition for meaningful transfer. Relatedly, despite random assignment, the two groups differed at baseline: mean CRRS was 95.67 vs. 108.00 points, mean age was 40.3 vs. 45.7 years, and both male participants were allocated to the comparison group. Regression toward the mean may therefore have contributed to the observed within-group changes, and gender is fully confounded with condition in this sample. The primary estimates reported above are unadjusted, and with a baseline difference of roughly 1.5 standard deviations on the CRRS, both the within-group estimates and the change-score contrasts remain susceptible to regression toward the mean. Adjusting for baseline CRRS, the between-group difference at four weeks was −4.08 points (95% CI [−13.07, 4.91]), consistent with the unadjusted change-score contrast. Moreover, although the comparison condition was matched in structure and role-play duration and was intended to impose comparable demands, cognitive and emotional load were not measured, and its scenarios did not involve emotional negotiation or service recovery, and thus the two conditions were not fully equivalent in socio-emotional demands. At the same time, the comparison scenarios (fraudulent calls) themselves required composure, verification, and assertive refusal—skills that overlap with several CRRS facets—so the between-group contrast was, in this respect, conservative with regard to customer-specific effects. Because the two conditions differed in visible content, participants may also have formed expectations about which training was intended to be useful for their work; the motivational difference at Day 8 and the comments describing the comparison scenarios as unrelated to the job are consistent with such an expectancy effect. The present design cannot separate this account from a difference in what the training conveyed.

The participant sample was also pre-dominantly female (10 of 12), reflecting the gender composition of the cooperating organization's customer support department rather than a deliberate sampling decision. While this composition is representative of the specific workplace, it limits generalizability to more gender-balanced populations. Examining moderating effects of gender and other demographic factors would require a sample recruited for that purpose.

The CRRS was developed by the authors, and its test–retest reliability has not been established. The magnitude of the observed change scores should therefore be interpreted with caution, and establishing that a change of this size reflects a change in responsiveness would require external criteria.

Furthermore, the feedback and performance scores in this system were generated by an LLM (GPT-4o) rather than validated against expert human ratings. Although the feedback content was co-designed with experienced call-center practitioners and iteratively refined for pedagogical alignment, the reliability and validity of the LLM-generated evaluations were not systematically assessed in this study, and the action classifier was not validated on spontaneous participant speech (Section 3.3). Establishing the concordance between LLM-generated and human-expert assessments would require a separate rating study with multiple blinded expert raters and a corpus of sessions large enough to estimate agreement; we therefore treat the feedback component as a design artifact whose pedagogical alignment was established through practitioner co-design, and we make no claim about its measurement validity. Moreover, deploying LLM-based evaluation in workplace training raises ethical considerations: if such scores were used for employee performance appraisal rather than formative learning, they could propagate biases inherent in LLM outputs and create inequitable outcomes. The present system was explicitly designed for self-directed learning, and participants were informed that training results would not affect workplace evaluations; nonetheless, establishing safeguards against evaluative misuse remains an important direction for responsible deployment.

The evaluation was conducted by the team that developed the system, which also selected the outcome measures, designed the comparison scenarios, authored the feedback content, and coded the open-ended responses (see Conflict of Interest Statement). Each of these choices could have shaped the results in ways the present design cannot isolate, and independent evaluation by researchers not involved in the system's development would provide stronger evidence.

Finally, the sample size limits what any single deployment can establish. The estimates reported here, together with their intervals, are intended to support sample-size planning for adequately powered evaluations of LLM-based role-play training.

### 6.5 Future work

The directions below concern the research programme rather than the present deployment, and each would require data that this study was not designed to collect. The present design cannot separate the contributions of the individual components: the LLM, the state-based dialogue control, the reflective feedback, the avatar, and the physiological display were deployed together. Identifying which of these carries the effect would require a factorial or component-based design in which components are added or withheld across conditions.

Future research could extend the present findings by incorporating individualized adaptation mechanisms that account for users' physiological states and personality traits. The real-time HR and RR waveforms displayed during training sessions may serve as a foundation for such mechanisms: although the present system used them only as reference information, future systems could employ these signals for stress-aware adaptive control that dynamically adjusts scenario intensity or feedback timing, maintaining a balance between emotional challenge and psychological safety. For example, when physiological arousal exceeds a certain level, the system could temporarily slow the escalation of the virtual customer's aggression or provide supportive feedback to facilitate emotional recovery before resuming the training. Such adaptive control would allow training to remain psychologically safe while maintaining sufficient challenge to foster skill development.

In addition, personality-related factors such as trait anxiety, emotional stability, or empathy may moderate the effectiveness of feedback and learning outcomes. Incorporating brief psychological assessments before training could enable the system to tailor interaction styles or feedback tones accordingly—for example, offering more structured guidance for participants with higher anxiety, and more autonomy-oriented feedback for those with greater self-efficacy. This direction aligns with the current findings showing variation in responsiveness and stress sensitivity among participants, contributing to a more personalized and equitable learning experience.

Finally, future research could explore adaptive scenario design. The present system employed fixed customer scenarios of moderate difficulty. Subsequent work could dynamically vary scenario complexity, escalation rate, or contextual detail based on users' progress and physiological indicators. Such fine-grained scenario control would enhance engagement and allow systematic examination of how stress level, motivation, and performance interact in human–AI role-play training environments.

## 7 Conclusion

This study presented an LLM-powered role-play training system that enables employees to practice managing aggressive customer interactions in a simulated setting designed to be psychologically safe. Through a 4-week field deployment, the largest point estimate for short-term motivation fell on the customer service group, and the within-group estimates for responsiveness and productivity in that group were medium-to-large. Qualitative accounts described greater attention to empathy, composure, and reflection, alongside reports of limited opportunity to apply the training. All confidence intervals include zero, and the comparison condition showed raw gains of comparable magnitude, so this deployment does not establish that the training improved the outcome scores or that any change was specific to customer-service content. What it does show is that the framework can be operated in a real workplace over four weeks, and that the largest between-group estimate fell on an immediate reaction-level measure whose content matches the training material, while the 4-week outcome estimates were near zero. Because such a measure can produce a large estimate whether or not the training transferred, evaluations of these systems should not rest on content-aligned reaction measures, and should verify that participants had opportunities to apply what was trained.

## Data Availability

The original contributions presented in the study are included in the article/Supplementary material, further inquiries can be directed to the corresponding author.

## Supplementary material

**Supplementary Data Sheet 1**

Scale items, dialogue architecture, example dialogues, pipeline verification, and original Japanese interface screenshots.
