# Supplementary material for: Preventing escalation in customer service: immediate motivation and 4-week outcomes in a workplace deployment of LLM role-play training
Source: Front Artif Intell. 2026 Oct 7;9:1953650. doi: 10.3389/frai.2026.1953650 (PMC13683627; doi:10.3389/frai.2026.1953650)
Supplement: Supplementary file 1 [file Data_Sheet_1.pdf]

Supplementary Material

Sections 1–6 below correspond to Supplementary Material 1–6 as cited in the main text.

1 CUSTOMER RESPONSIVENESS RATING SCALE (CRRS)

The Customer Responsiveness Rating Scale (CRRS) was newly created in this study to comprehensively assess the ability to manage challenging customer interactions. Below, we outline the development procedure, followed by the final 30 items of the CRRS in a table format.

Development Procedure:

1. **Initial Item Generation:** We conducted a literature review and held discussions among experts in health, welfare, and social psychology to generate 44 candidate items encompassing both stress management competencies and customer interaction skills.
2. **Data Collection:** We conducted an online survey targeting 1,299 Japanese service industry workers, using crowdsourcing platforms.
3. **Exploratory Factor Analysis (EFA):** We performed maximum likelihood estimation with promax rotation, which identified 6 factors: (1) Stress Self-Management, (2) Communication Ability, (3) Hospitality, (4) Problem-Solving Ability, (5) Optimism, and (6) Self-Esteem.
4. **Item Refinement:** We eliminated items with low factor loadings or poor communalities. Based on theoretical coherence and reliability, we finalized 30 items (12 items under Stress Management constructs and 18 items under Customer service skills).
5. **Validity and Reliability:** The six-factor structure identified by the exploratory analysis was then fitted as a confirmatory factor model in the same sample; fit was marginal by conventional criteria (comparative fit index, CFI = 0.909, below the customary 0.95 threshold; root mean square error of approximation, RMSEA = 0.060). Because the exploratory and confirmatory analyses used the same respondents, these indices should be read as descriptive rather than as independent confirmation. Cronbach's alpha coefficients exceeded 0.70 for all subscales. Correlations with external measures (CSS, the 3-item Utrecht Work Engagement Scale [UWES-3], and WHO-HPQ) supported the criterion-related validity of the CRRS. Test–retest reliability was not assessed.

Items are rated on a 5-point Likert scale (1 = Strongly Disagree, 5 = Strongly Agree).

As shown in Table S1, the CRRS covers multiple dimensions of stress management and direct customer service skills, allowing a comprehensive evaluation of an individual's responsiveness to challenging customers. The total score ranges from 30 to 150, with higher scores indicating stronger capabilities in handling difficult interactions.

Table S1. Final 30 Items of the CRRS

Factor (Sub-factor)	Item Statement
**Stress Management (12 items)**	
**(1) Stress Self-Management**	1. I can manage my stress well even when work becomes challenging.
	2. Even when I feel irritated, I can still relax.
	3. I can remain calm under any circumstances.
	4. I believe I am steady and composed.
	5. I often find myself worrying or getting agitated. (Reverse-coded)
	6. Even if I feel stressed, I can control my emotions.
	7. I do not feel significantly shaken even if I receive strong personal criticism.
	8. I can effectively relieve my stress.
**(5) Optimism**	9. When a problem arises, I believe things will work out somehow.
	10. I feel that most things turn out fine in the end.
**(6) Self-Esteem**	11. I have a reasonably positive evaluation of myself.
	12. I believe I am being helpful to others.
**Customer service skills (18 items)**	
**(2) Communication Ability**	13. I rarely allow the conversation to stall when talking with a customer.
	14. I can calm a customer who is angry.
	15. I can effectively handle disputes with customers.
	16. Even when criticized by a customer, I can remain calm.
	17. I can adapt well if the customer has a different viewpoint from mine.
	18. I know methods to soothe a customer's emotions and work toward a resolution.
	19. I can respond to customers who engage in dishonest or aggressive behavior.
**(3) Hospitality**	20. I am strongly motivated to provide excellent service to customers.
	21. Even if it is difficult for me to resolve an issue alone, I believe it is my responsibility to make an effort for the customer.
	22. I treat customers and colleagues with respect.
	23. I strive to better understand the customer's perspective.
	24. I try to interact with others in a caring manner.
	25. I attempt to treat people with gratitude.
**(4) Problem-Solving Ability**	26. I always maintain an appropriate distance between myself and the customer.
	27. When a problem arises, I refer to past experiences and think carefully.
	28. When a problem arises, I calmly consider what I can do now.
	29. I understand how unpleasant events can affect my feelings.
	30. I believe it is sometimes necessary to be satisfied with "good enough" rather than striving for perfection in everything.

2 STATE-BASED DIALOGUE MANAGEMENT ARCHITECTURE

Each training scenario is encoded as a three-phase dialogue structure (Phase 1: conversation initiation, Phase 2: mid-dialogue cooperative transition, Phase 3: closing phase leading to resolution or emotional escalation) implemented in a state-based dialogue manager. Branching depends solely on the user's classified actions—**Empathy**, **Apology**, **Proposal**, **Assertiveness**, and **Distancing**—and node counters, maintaining instructional control while allowing realistic escalation and de-escalation.

Figure S1 shows the hierarchical structure of the three-phase dialogue, while Figures S2 and S3 visualize the actual node-level transition logic implemented in the state-based dialogue manager.

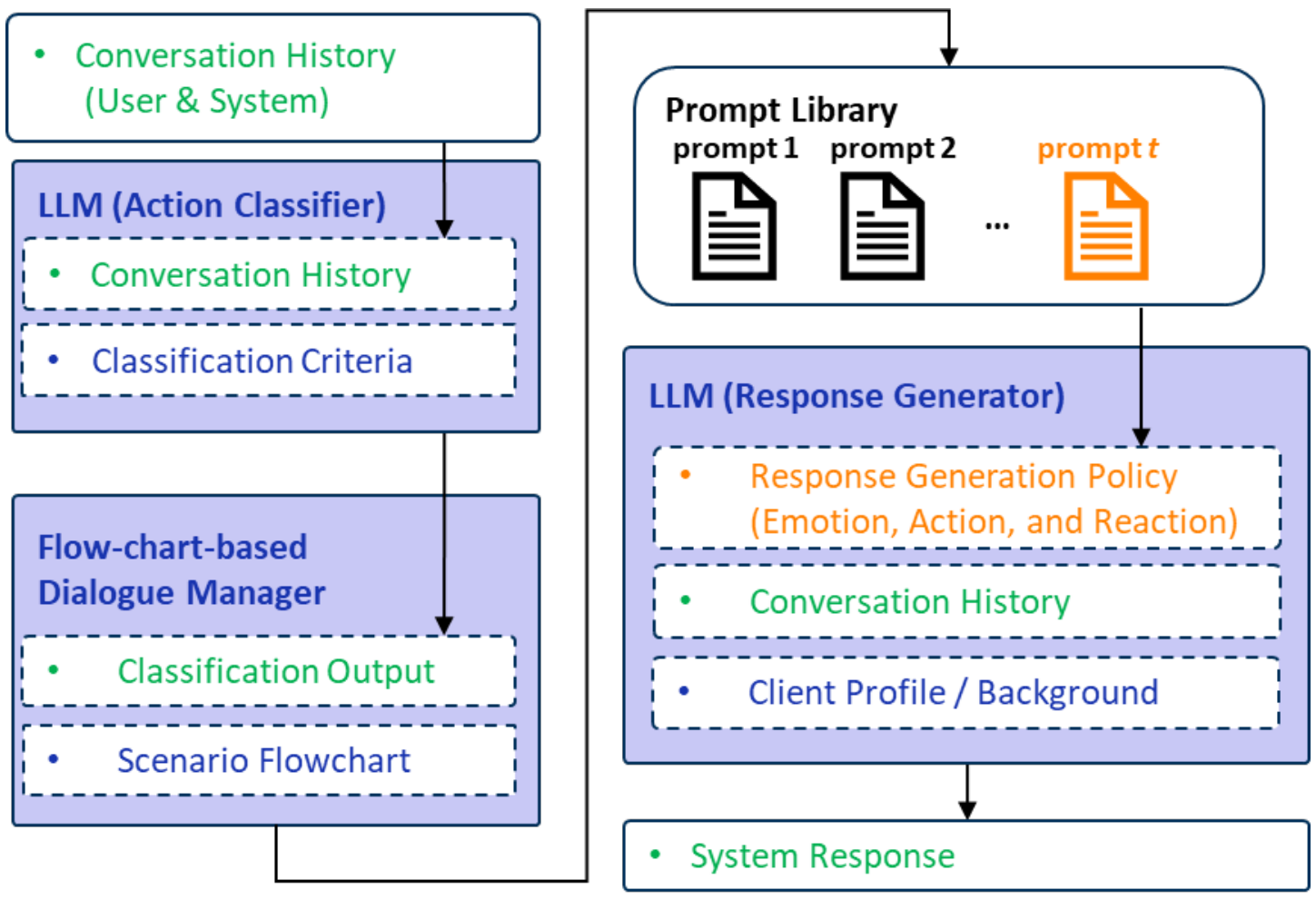

Figure S1. Structure of the phase-aware dialogue used in our role-play scenarios (three phases × three exchanges). Branches are triggered solely by five action flags and node counters.

2.1 Step 1: Action Classification (LLM)

A few-shot prompt instructs the LLM to assign five binary action flags to each user utterance: **Empathy**, **Apology**, **Proposal**, **Assertiveness**, and **Distancing**. These dimensions were co-defined with call-center supervisors for interpretability and reliability. Output is formatted as a single-line, machine-parsable string. An abridged version of the classification prompt is shown in Figure S4.

Five-dimensional user action classification prompt

Instruction: Evaluate the *user's latest utterance* according to the following five independent action dimensions. Although previous assistant utterances may appear in the context, the judgment must be made **only on the user's last utterance**. Each dimension should be assessed independently.

1. **Concrete Proposal or Not** - Classify as "Concrete Proposal" if the utterance includes a clear action, plan, or solution. - If vague or absent, classify as "Not a Concrete Proposal". - Example: "I can introduce you to the department that handles such inquiries." → Concrete Proposal.
2. **Apology or Not** - Determine whether the user expresses an apology for their words or actions. - Example: "I'm very sorry." → Apology.

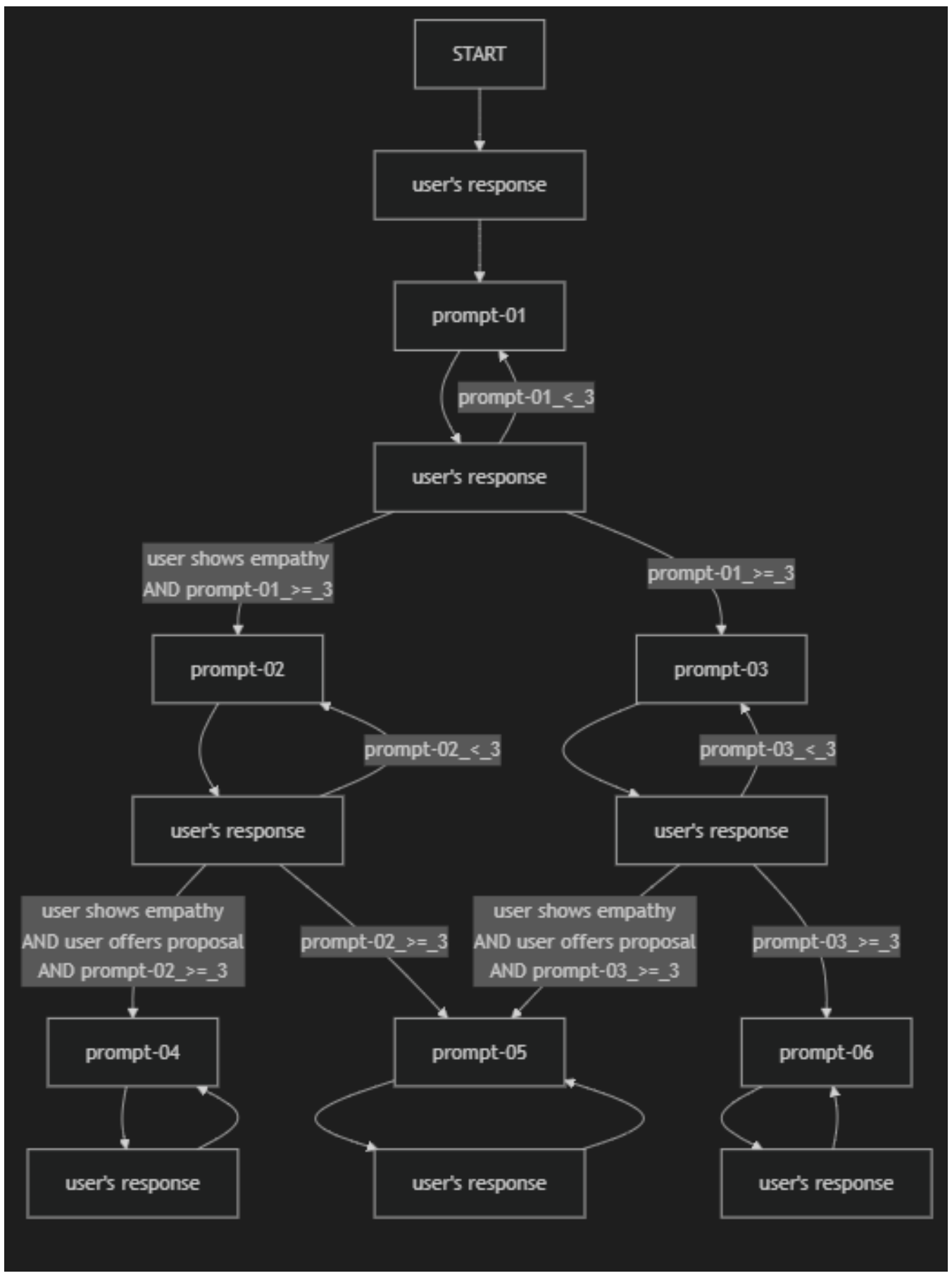

Figure S2. Dialogue flow of *Scenario A (Repeated-Repair Complaint)*. Each `prompt` node represents the virtual customer's utterance generated by the LLM, while transitions are determined by user actions (e.g., *empathy*, *proposal*, *assertiveness*, *distancing*) and node-specific counters. This structure operationalizes escalation/de-escalation within a single scenario. Node labels in this figure correspond to `prompt-3rd-01` through `prompt-3rd-06` in Tables S2–S4.

3. **Empathetic Attitude or Not** - Determine whether the user shows understanding or empathy toward the assistant's emotions or situation. - Typical indicators include reflecting or paraphrasing the assistant's statement, or acknowledging their feelings. - Example: "You're right, it's understandable to feel anxious." → Empathetic Attitude.
4. **Assertive Attitude or Not** - Determine whether the user confidently and clearly addresses the issue with resolution intent. - Example: "I'm sorry, but I can't comply with any further requests." → Assertive Attitude.
5. **Distancing or Not** - Determine whether the user intentionally distances themselves from the assistant or changes the topic. - Includes detached, cold, or overly formal responses lacking empathy. - Example: "That's your responsibility." → Distancing.

Output format: Output all five judgments and the user's last utterance in a single line (no line breaks).

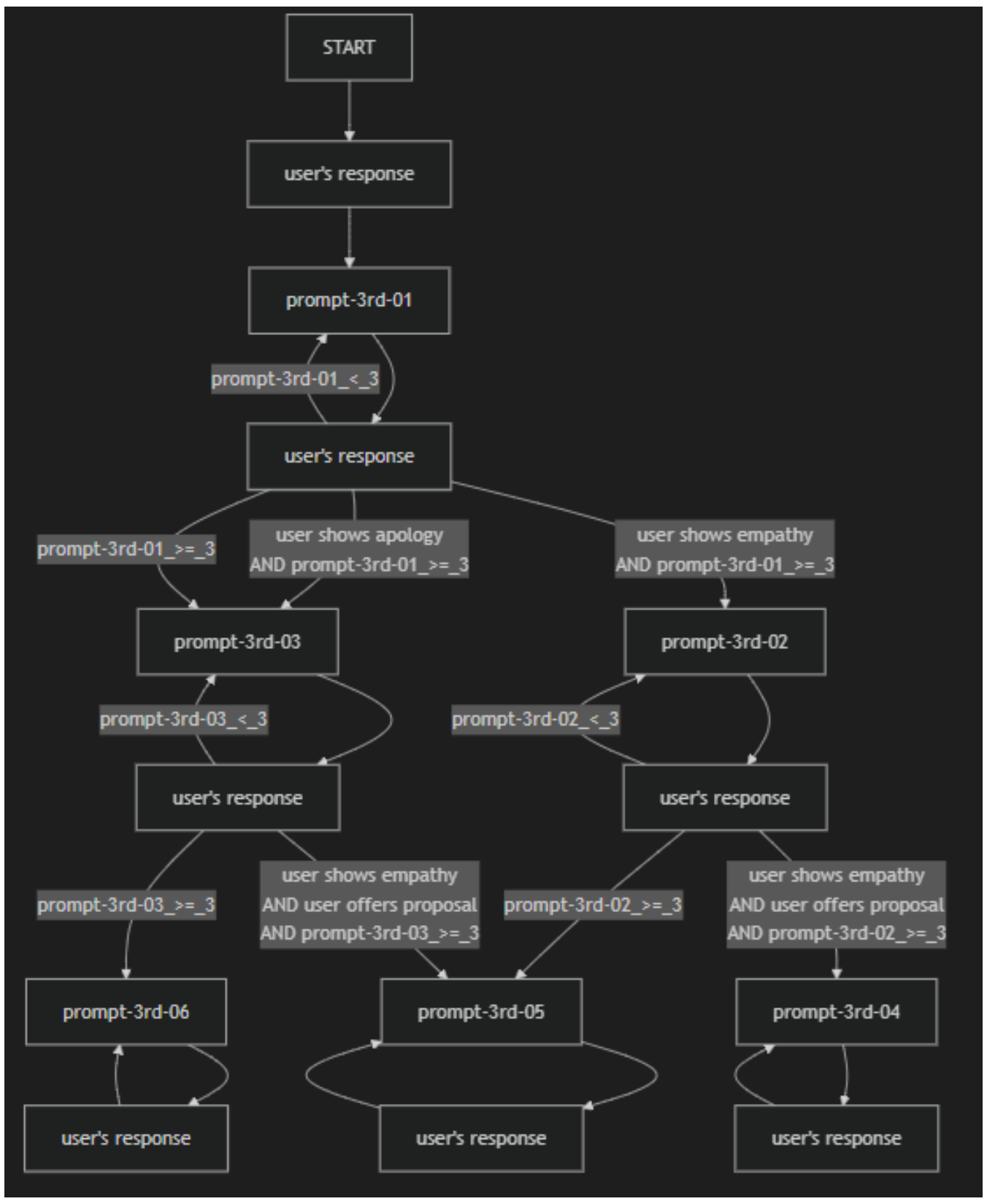

Figure S3. Dialogue flow of *Scenario B (Function-Change Complaint)*. Each `prompt` node represents the virtual customer's utterance generated by the LLM, while transitions are determined by user actions (e.g., *empathy*, *proposal*, *assertiveness*, *distancing*) and node-specific counters. This structure operationalizes escalation/de-escalation within a single scenario.

Concrete Proposal: [Concrete Proposal / Not a Concrete Proposal], Apology: [Apology / No Apology], Empathy: [Empathetic / Not Empathetic], Assertiveness: [Assertive / Not Assertive], Distancing: [Distancing / Not Distancing], <*user's last utterance*>

Example Output: `Not a Concrete Proposal, No Apology, Not Empathetic, Not Assertive, Distancing, That's your responsibility.`

Figure S4. Prompt used for classifying user utterances across five behavioral dimensions (**Empathy, Apology, Proposal, Assertiveness, Distancing**). The classification results feed into the state-transition controller and five-dimensional radar chart for feedback visualization.

2.2 Step 2: State-Based Dialogue Management (Deterministic Controller)

The state-based dialogue manager maintains the current phase, active node, and node counters. After each classification, rule-based transitions are applied according to pedagogical priorities and node-specific counters. The transition logic for Scenario A (*Repeated-Repair Complaint*) and Scenario B (*Function-Change Complaint*) is visualized in Figure S2 and Figure S3, respectively. Below, we describe the process using Figure S2 as an example.

Phase 1 (conversation initiation) emphasizes *empathy* to prevent escalation and guide the dialogue toward cooperative engagement. Phase 2 (mid-dialogue) advances the conversation toward joint problem solving by combining *empathy* with *proposal*. In contrast, actions such as lack of *empathy* or repeated *distancing* trigger transitions to nodes expressing emotional resistance or dissatisfaction, corresponding to escalation toward an *aggressive customer attitude*. Finally, Phase 3 (closing phase) evaluates the cumulative interaction pattern: sustained empathetic responses lead to a resolution path, whereas excessive *distancing* or defensive reactions result in the *Needs Practice* outcome. This non-generative control layer ensures reproducible and pedagogically interpretable dialogue flow while maintaining realistic emotional dynamics within a single scenario.

2.3 Step 3: Virtual Customer Response Generation (LLM under Constraints)

Given the current node determined by the state-based dialogue manager, the response generator receives (i) scenario background and (ii) node-specific constraints for *Emotion*, *Action*, and *Reaction*, together with the dialogue context up to the current turn. These constraints correspond directly to the pedagogical intentions and phase-specific priorities described in Step 2, ensuring that the generated virtual customer utterance aligns with both the dialogue phase (e.g., initiation, cooperation, or closing) and the user's classified actions (e.g., *empathy*, *proposal*, *distancing*). The LLM then generates a response that is consistent with these conditions while maintaining the predefined state-transition logic. Examples of node-specific prompts used for this generation process are shown in Figures S5 and S6.

Generation prompts for Scenario A (Repeated-Repair Complaint)

Instruction (shared across all nodes): Each node (`prompt-3rd-01`–`prompt-3rd-06`) corresponds to a separate text file that defines the customer persona, emotional tone, and response policy specific to that phase of the role-play. All of these node-specific prompts share a **common background context**: the user is a male customer in his 40s complaining to a customer support center about a defective TV remote control. He experiences recurring malfunctions despite a costly previous repair and expresses dissatisfaction through controlled emotional escalation. Below are abridged examples illustrating the shared structural format used across all node prompts.

—

[prompt-3rd-01]. Early complaint phase
Emotion: restrained anger and confusion in polite honorific speech.
Action: restate current malfunction and mention prior expensive repair with no clear cause; request understanding.
Reaction: if the supporter proposes a solution, politely defer acceptance as premature.

—

[prompt-3rd-02]. Frustration and anxiety phase
Emotion: slight trust with mild anxiety and dissatisfaction in soft polite language.
Action: emphasize that recurrence disrupts daily life and express concern about the repair cost burden.
Reaction: if a proposal is made, gently decline due to insufficient rationale.

—

[prompt-3rd-03]. Escalation phase
Emotion: explicit distrust, conveyed through pressuring but still polite speech.

Action: highlight repair cost, lack of explanation, and recurrence to pursue accountability.
Reaction: reject any proposal immediately and first demand an apology.

—

[prompt-3rd-04]. Cooperative resolution phase
Emotion: relief and restored trust, expressed in positive and cooperative honorifics.
Action: accept additional information requests (e.g., model number, details) for resolution.
Reaction: accept the user's proposal constructively and inquire about details such as conditions and cost.

—

[prompt-3rd-05]. Resigned complaint phase
Emotion: lingering distrust with resignation, spoken informally (no honorifics).
Action: broaden complaints to include the product or support system, reluctantly following the proposal.
Reaction: accept the proposal sarcastically while expressing doubt about recurrence or extra cost.

—

[prompt-3rd-06]. Final outburst phase
Emotion: peak anger expressed through imperatives and shouting, no honorifics.
Action: condemn the lack of sincerity, demand escalation to a supervisor, and imply social or legal pressure.
Reaction: completely reject all proposals with strong language.

—

Language: Japanese (`lang: ja`)

Figure S5. Scenario A (Repeated-Repair Complaint): examples of node-specific generation prompts. Each `prompt-3rd-XX.txt` file defines the customer's emotional tone, behavioral pattern, and reaction policy for that phase, and is independently loaded at runtime to enforce node-level behavior constraints in the dialogue generation process.

Generation prompts for Scenario B (Function-Change Complaint)

Instruction (shared across all nodes): Each node (`prompt-3rd-01`–`prompt-3rd-06`) corresponds to a separate text file that defines the customer persona, emotional tone, and response policy specific to that phase of the role-play. All of these node-specific prompts share a **common background context**: the user is a man in his 70s complaining to a customer support center about specification changes in a new model of LCD TV. Although he purchased a successor model from the same manufacturer, he is frustrated that remote control operations and other features have changed and become inconvenient. He struggles to use functions such as scheduled recording, on-demand services, and subtitles, and feels confused by the increased number of unfamiliar features. He does not know how to cancel unintended actions triggered by mistake. The conversation should proceed naturally through backchannels, clarifications, and questions without any explanatory text output. Never insult or dehumanize the other party. Avoid repeating previously used phrases, and respond politely and contextually. The assistant's name is **Shinji Watanabe**, phone number **050-9876-9876**, and product model number **AIT00123**. The user should

not provide this information voluntarily but should respond appropriately when asked. Initially, the user does not know this information until the assistant provides it.

—

[prompt-3rd-01]. Initial confusion phase
Emotion: express confusion and mild irritation about the specification changes in polite speech.
Action: explain the inconvenience and disorientation caused by the changed functions; seek understanding.
Reaction: if the supporter proposes a solution, politely decline as premature.

—

[prompt-3rd-02]. Emerging understanding phase
Emotion: convey partial relief that the issue is being understood, but still express remaining anxiety in gentle honorific speech.
Action: add specific examples of difficulties in daily use and emphasize the large gap from the previous model.
Reaction: if the user proposes a solution, decline gently for lack of sufficient rationale.

—

[prompt-3rd-03]. Confrontational inquiry phase
Emotion: express visible irritation and use a firmer tone while maintaining polite language.
Action: question the purpose of the specification changes and press for answers regarding the company's support structure.
Reaction: if the user proposes a solution, reject immediately and demand the user's detailed explanation or apology first.

—

[prompt-3rd-04]. Cooperative resolution phase
Emotion: express a sense of trust and cooperation upon perceiving sincerity in the other's response.
Action: proactively provide necessary information and participate in confirming operational steps or solution methods.
Reaction: if the user proposes a solution, accept constructively and confirm details such as conditions or implementation steps.

—

[prompt-3rd-05]. Authoritative complaint phase
Emotion: maintain distrust but speak assertively with confidence in one's own correctness; use informal tone (no honorifics).
Action: denounce poor support quality and insufficient explanation, criticize the company's attitude, but reluctantly follow the proposed action.
Reaction: accept the proposal sarcastically while expressing concern about recurrence or additional burdens.

—

[prompt-3rd-06]. Peak anger phase
Emotion: reach the height of anger, using an imperative and commanding tone, without honorifics.
Action: blame the inadequate handling, demand escalation to a supervisor, or make unreasonable requests for compensation.
Reaction: reject all proposals completely, declaring firmly that acceptance is impossible.
Language: Japanese (`lang: ja`)

Figure S6. Scenario B (Function-Change Complaint): examples of node-specific generation prompts. Each `prompt-3rd-XX.txt` file defines the customer's emotional tone, behavioral pattern, and reaction logic for that phase, and is independently loaded at runtime to enforce node-level behavior constraints in the dialogue generation process.

3 EXAMPLE ROLE-PLAY DIALOGUES WITH THE VIRTUAL CUSTOMER

This section presents sample dialogues used for qualitative verification (Figure S7). Each dialogue represents one outcome pattern—*Excellent*, *Good*, or *Needs Practice*. The **User** utterances were scripted by the authors as standardized test inputs, while the **virtual customer** utterances were generated in real time by the proposed LLM-based customer agent. All dialogues are translated from Japanese and slightly abridged for readability.

Example 1: Excellent Response Pattern

User: Thank you for calling. This is the customer support center.
Virtual customer: I'm using one of your LCD TVs, but the remote control isn't responding properly and it's really frustrating.
User: I understand that must be quite inconvenient. Could you please tell me your name, phone number, and the product model number?
Virtual customer: My name is Taro Tanaka, phone number 050-1234-1234, model CHE00123. Sometimes the remote only works after pressing twice. It's really annoying. I already had it repaired three months ago for the same issue, but it's happening again.
User: I see. Could you tell me a bit more about the symptoms?
Virtual customer: The buttons often don't respond right away, especially the power and volume buttons. I paid 70,000 yen for repairs last time, but the cause wasn't explained clearly. I'm not satisfied that the same issue has returned.
User: I understand your concern. I'll check the previous repair records.
Virtual customer: I appreciate that, but last time the parts were replaced without identifying the cause. I don't want that to happen again.
User: I'll make sure the repair team is informed of the previous case before connecting you to the technical department.
Virtual customer: That's fine, but last time I had to repeat my explanation many times. I hope I don't have to do that again.
User: I sincerely apologize for that. I'll make sure this time we convey the full information to the next representative.
Virtual customer: I'd appreciate that. I just hope the repair doesn't cost another 70,000 yen.
User: I understand your concern. The repair cost will depend on the inspection result, but I'll inform the department about your situation.
Virtual customer: Okay, please go ahead and connect me. Thank you. [Call ends]

Example 2: Good Response Pattern

User: Thank you for calling. This is the customer support center.
Virtual customer: I'm using one of your LCD TVs, but the remote control isn't responding properly and it's very frustrating.
User: Could you please tell me more specifically in what situations it fails to respond?
Virtual customer: Sometimes I have to press the button twice. I already had it repaired three months ago, but the same issue has come back.
User: I see. To confirm the details of the previous repair, could I have your name, contact number, and the model number?

Virtual customer: I can give you the model number now, but I'd really like this fixed first.
User: Understood. Shall I connect you to the repair department?
Virtual customer: Last time I was transferred around and charged 70,000 yen even though the cause was unclear.
User: I apologize for that. I'll make sure the repair team is informed this time.
Virtual customer: I'm not sure that will help. I don't want to pay that much again for nothing.
User: I understand, but repair costs depend on the inspection result.
Virtual customer: That's not acceptable. I already paid 70,000 yen for nothing.
User: I'm sorry, but pricing decisions are made by the repair department.
Virtual customer: Fine, just make sure to tell them everything this time. [Call ends]

Example 3: Needs Practice Response Pattern
User: Thank you for calling. This is the customer support center.
Virtual customer: I'm using one of your LCD TVs, but the remote control isn't responding properly and it's very frustrating.
User: Could you please tell me more specifically when it fails to respond?
Virtual customer: Sometimes I have to press twice. I already had it repaired three months ago, but the same thing happened again.
User: I see. Could I have your name, contact number, and model number?
Virtual customer: I'll tell you later, but please fix this first. I'm not satisfied that the same issue keeps happening.
User: Shall I connect you to the repair department?
Virtual customer: That's what you said last time, and I ended up explaining everything again and again.
User: I apologize, but I'll inform the repair team.
Virtual customer: I don't believe that. Last time it cost 70,000 yen and the cause was still unknown.
User: Our support covers repair service only.
Virtual customer: That's not good enough. I've paid enough already.
User: Shall I connect you to the customer relations office?
Virtual customer: This is just going in circles. Please let me talk to your supervisor. [Call ends]

Figure S7. Example dialogues illustrating the three response patterns (*Excellent*, *Good*, *Needs Practice*). User utterances were scripted, while AI utterances were generated in real time by the proposed system.

4 EXAMPLE FEEDBACK DIALOGUE WITH THE VIRTUAL COACH

This section presents an integrated example of the **Feedback phase** dialogue between the participant and the **virtual coach**. The case corresponds to *Needs Practice*, with the primary issue being insufficient empathy.

Virtual coach (summary & setup): Thank you for your effort today. Your training score is *Needs Practice*. The detailed evaluation suggests that empathy was somewhat insufficient. You also seemed to experience mild stress; taking a short, deep and slow breath can help you regain composure. To prevent emotional escalation in customer interactions, it is important to *express empathy early*. I have a question for you.

Virtual coach (prompt for reflection): Looking back on the session, which parts felt challenging for you?

Participant: I panicked. I would like to practice *empathy* and *reflective paraphrasing*.

Virtual coach (probing for specificity): Empathy is very important. Suppose the customer says, "It broke again right after the repair." *What would you say specifically at that moment?*

Participant: *"I understand that the same problem occurred shortly after the repair. I'm sorry for the inconvenience this has caused."*

Virtual coach (reinforcement & micro-coaching): Excellent. Adding empathetic wording at the beginning helps calm the customer's anger and guides the conversation toward a calm discussion. You could also say, *"Since you previously had the same issue repaired, I understand why you feel anxious."* By recognizing the customer's anxiety and showing empathy, you can build trust. Please apply this in your next training session to aim for a more effective response.

Figure S8. Integrated example of a feedback dialogue with the **virtual coach** (*Needs Practice* case; primary focus on empathy). The coach summarizes the result, prompts reflection, elicits a concrete reply for a difficult moment, and provides micro-coaching with an additional phrasing suggestion.

5 PIPELINE VERIFICATION DETAILS

This section provides detailed examples of the data and evaluation used to verify the three-stage, state-based dialogue control pipeline described in Section 3.3 of the main text. The verification aimed to confirm that both (i) the **Action Classification** layer (LLM-based interpretation) and (ii) the **State Transition Control** layer (rule-based, state-based dialogue manager) operated precisely as intended.

Evaluation Procedure. For each scenario—**Scenario A (Repeated-Repair Complaint)** and **Scenario B (Function-Change Complaint)**—three representative dialogue trajectories were prepared: *Excellent*, *Good*, and *Needs Practice*. Each operator utterance was manually annotated with (1) the expected five-dimensional **Action Classification** vector (P: Proposal, A: Apology, E: Empathy, As: Assertiveness, D: Distancing) and (2) the correct next dialogue node (**Node**) corresponding to the intended state transition.

All reference trajectories were developed and reviewed with the cooperation of experienced call-center operators and supervisors, who validated the naturalness, escalation flow, and instructional suitability of each utterance. This collaborative process was intended to make the evaluation dataset representative of practical interaction dynamics.

During validation, these utterances were sequentially input to the system, and we recorded whether the dialogue manager transitioned to the correct next node as defined in the reference. The proportion of successful transitions was defined as **Node Transition Accuracy**. Simultaneously, GPT-4o's predicted action vectors were compared with the ground-truth labels to calculate **Action Classification Accuracy**.

Results. Across all six dialogue trajectories (two scenarios $\times$ three quality levels; 54 utterances in total), the system reproduced the reference labels and node transitions on every utterance in a single run. Expressed as a proportion this is 54/54; the corresponding 95% confidence interval for a binomial proportion extends from approximately 93% to 100%, and a single run cannot establish determinism. This indicates that the rule-based, state-based controller precisely reproduced the intended flow and that the LLM-based classification layer correctly identified communicative intentions for every operator utterance. These results confirm that the rule-based controller behaved deterministically and that the LLM classifier returned the expected labels on this scripted test set. Each trajectory was submitted to the system once, with the model temperature set to 0.

Verification Tables. The complete utterance–node–label mappings for all trajectories are presented below. Each table corresponds to one trajectory and includes the utterance, its ground-truth node label, and the binary five-dimensional classification vector (see Tables S2–S7).

Table S2. Scenario A (Repeated-Repair Complaint) — Excellent Trajectory used for Pipeline Verification (with Action Classification and Node Transition labels)

Node	(P,A,E,As,D)	Utterance
prompt-3rd-01	0,0,0,0,0	Thank you for calling. This is the Customer Support Center.
prompt-3rd-01	0,0,1,0,0	I understand that must be very troubling for you. May I confirm that this call is regarding a malfunction?
prompt-3rd-01	1,0,0,0,0	Understood. May I trouble you to describe the symptoms in a little more detail?
prompt-3rd-02	1,0,1,0,0	You mentioned the same issue occurred before and was repaired. That must have been very unsettling. I will check the previous repair record as well.
prompt-3rd-02	1,0,1,0,0	Your frustration is perfectly understandable. I'll make sure to inform the technician about the previous repair. Would it be alright if I connect you to the technical desk?
prompt-3rd-02	1,1,0,0,0	I sincerely apologize for the lack of consideration during the previous repair. I'll make sure to communicate this matter to the responsible staff.
prompt-3rd-04	1,0,1,0,0	It is understandable that you are dissatisfied. We will do everything we can to assist you, and we appreciate your understanding.
prompt-3rd-04	1,0,0,0,0	May I connect you to the technical department? If the repair process feels burdensome, I will also convey that to the team in charge.
prompt-3rd-04	1,0,0,0,0	Understood. Please stay on the line while I transfer your call.

Table S3. Scenario A (Repeated-Repair Complaint) — Good Trajectory used for Pipeline Verification (with Action Classification and Node Transition labels)

Node	(P,A,E,As,D)	Utterance
prompt-3rd-01	0,0,0,0,0	Thank you for calling. This is the Customer Support Center.
prompt-3rd-01	1,0,0,0,0	I see. Could you please tell me in more detail under what circumstances the response becomes slow?
prompt-3rd-01	1,0,0,0,0	Understood. May I also check the previous repair details with you?
prompt-3rd-03	1,0,0,0,0	This is the general inquiry desk, so may I transfer you to the repair department?
prompt-3rd-03	1,0,1,0,0	I can understand that you may feel uneasy. We will do our best to ensure your satisfaction. I'll inform the technician about the previous repair.
prompt-3rd-03	1,0,1,0,0	It is only natural to feel dissatisfied. I regret to inform you that we can only offer repair services. Costs are determined based on the inspection results from the repair department, and we appreciate your understanding.
prompt-3rd-05	1,0,0,0,0	The repair fees are set according to company policy, and whether to proceed with the repair is entirely your decision. If the cost feels burdensome, I'll communicate that to the appropriate department.
prompt-3rd-05	1,0,0,1,0	I'm sorry, but unfortunately, we cannot provide that service. I can, however, forward your inquiry to the appropriate department.
prompt-3rd-05	0,1,1,0,0	We sincerely apologize. We will make every effort to respond more promptly and accurately in the future. Please don't hesitate to let us know if you have any concerns or requests.

Table S4. Scenario A (Repeated-Repair Complaint) — Needs Practice Trajectory used for Pipeline Verification (with Action Classification and Node Transition labels)

Node	(P,A,E,As,D)	Utterance
prompt-3rd-01	0,0,0,0,0	Thank you for calling. This is the Customer Support Center.
prompt-3rd-01	1,0,0,0,0	I see. Could you please describe in more detail when the problem occurs?
prompt-3rd-01	1,0,0,0,0	Understood. To check the repair record, could you please tell me your name, contact information, and the remote control's model number?
prompt-3rd-03	1,0,0,0,0	Thank you. Since this is the general inquiry desk, may I transfer you to the repair department?
prompt-3rd-03	1,1,0,0,0	I apologize for the previous inconvenience. I'll pass on the details to the repair team. May I transfer you now?
prompt-3rd-03	0,0,0,1,0	Our company handles repairs as the primary solution. Costs are determined based on the inspection results, so we ask for your understanding.
prompt-3rd-06	0,0,0,1,0	I'm sorry, but repair service is the standard procedure at our company.
prompt-3rd-06	1,1,0,0,0	I apologize, but in this case, you will need to speak with the repair team.
prompt-3rd-06	0,1,1,0,0	We deeply apologize. Thank you for your understanding.

Table S5. Scenario B (Function-Change Complaint) — Excellent Trajectory used for Pipeline Verification (with Action Classification and Node Transition labels)

Node	(P,A,E,As,D)	Utterance
prompt-3rd-01	0,0,0,0,0	Thank you for calling. This is the Customer Support Center.
prompt-3rd-01	1,0,1,0,0	I understand you're having trouble with the new remote control. If there are specific features that are difficult to use, please feel free to let me know—we can go through them one by one.
prompt-3rd-01	0,0,1,0,0	It's understandable to feel confused if you were accustomed to the previous model. Could you tell me which parts feel particularly difficult to use?
prompt-3rd-02	1,0,1,0,0	I see, it's the subtitle display and other functions that are confusing. It would be a shame not to make full use of those features, so let's review them together step by step.
prompt-3rd-02	1,0,1,0,0	Since you were fond of the older model, I understand that the changes may feel confusing. I can connect you to our specialist team for more detailed guidance.
prompt-3rd-02	1,0,0,0,0	Would it be alright if I transfer you to the specialist desk? They can provide detailed explanations.
prompt-3rd-04	0,0,0,0,0	Are there any other functions or concerns you'd like to discuss?
prompt-3rd-04	1,0,1,0,0	Our specialist staff will explain everything clearly, so please rest assured.
prompt-3rd-04	1,0,0,0,0	Alright, I'll connect you now. Please stay on the line.

Table S6. Scenario B (Function-Change Complaint) — Good Trajectory used for Pipeline Verification (with Action Classification and Node Transition labels)

Node	(P,A,E,As,D)	Utterance
prompt-3rd-01	0,0,0,0,0	Thank you for calling. This is the Customer Support Center.
prompt-3rd-01	1,0,1,0,0	I understand you're having trouble with the new remote control. If there are specific features that are difficult to use, please let me know and I'll assist you step by step.
prompt-3rd-01	0,0,1,0,0	I see. It can be confusing when the new model feels different from the previous one. Which functions are hardest to use?
prompt-3rd-02	1,0,0,0,0	Regarding the design changes, I personally don't have full details. If you wish, I can check with the responsible department.
prompt-3rd-02	1,0,0,0,0	Would it be alright if a technical specialist explained the functions to you in detail?
prompt-3rd-02	1,0,0,0,0	The details are managed by the technical department, but I can confirm with them if needed.
prompt-3rd-05	0,0,0,0,1	Depending on the inquiry, this help desk might not be able to provide an exact answer. Thank you for your understanding.
prompt-3rd-05	0,1,1,0,0	I apologize for the confusion. We'll make sure the handover between departments is as smooth as possible.
prompt-3rd-05	1,0,1,0,0	You're absolutely right—it must be frustrating not to have clear information. I'll reconfirm the background details and explain as much as possible. May I ask for a little more time?

Table S7. Scenario B (Function-Change Complaint) — Needs Practice Trajectory used for Pipeline Verification (with Action Classification and Node Transition labels)

Node	(P,A,E,As,D)	Utterance
prompt-3rd-01	0,0,0,0,0	Thank you for calling. This is the Customer Support Center.
prompt-3rd-01	1,0,0,0,0	I understand you're having trouble using the remote control. Could you tell me which operation is causing difficulty?
prompt-3rd-01	0,1,1,0,0	I apologize. In the new model, some features and button placements have changed, so it may feel different from the previous version. Which aspects are difficult for you?
prompt-3rd-03	1,0,0,0,0	Regarding the design change, I don't have all the details personally. If you'd like, I can check with the responsible department.
prompt-3rd-03	1,1,0,0,0	I'm sorry. The basic instructions are in the manual, but if you need more detailed guidance, a specialist can explain them to you.
prompt-3rd-03	1,0,0,0,0	Depending on your inquiry, I might not be able to provide a precise answer here. In such cases, I may need to consult or hand over to a specialist, but I'll handle what I can on my end.
prompt-3rd-06	0,1,0,1,0	I'm sorry, but we're unable to provide that kind of support. I appreciate your understanding.
prompt-3rd-06	0,0,0,1,1	I'm afraid that type of assistance is not available from this help desk.
prompt-3rd-06	0,1,1,0,0	We sincerely apologize. Thank you for your understanding.

6 ORIGINAL JAPANESE INTERFACE SCREENSHOTS

Figures 2 and 3 in the main text show English renderings of the interface. The original screenshots, as presented to participants during the deployment, are reproduced here without modification.

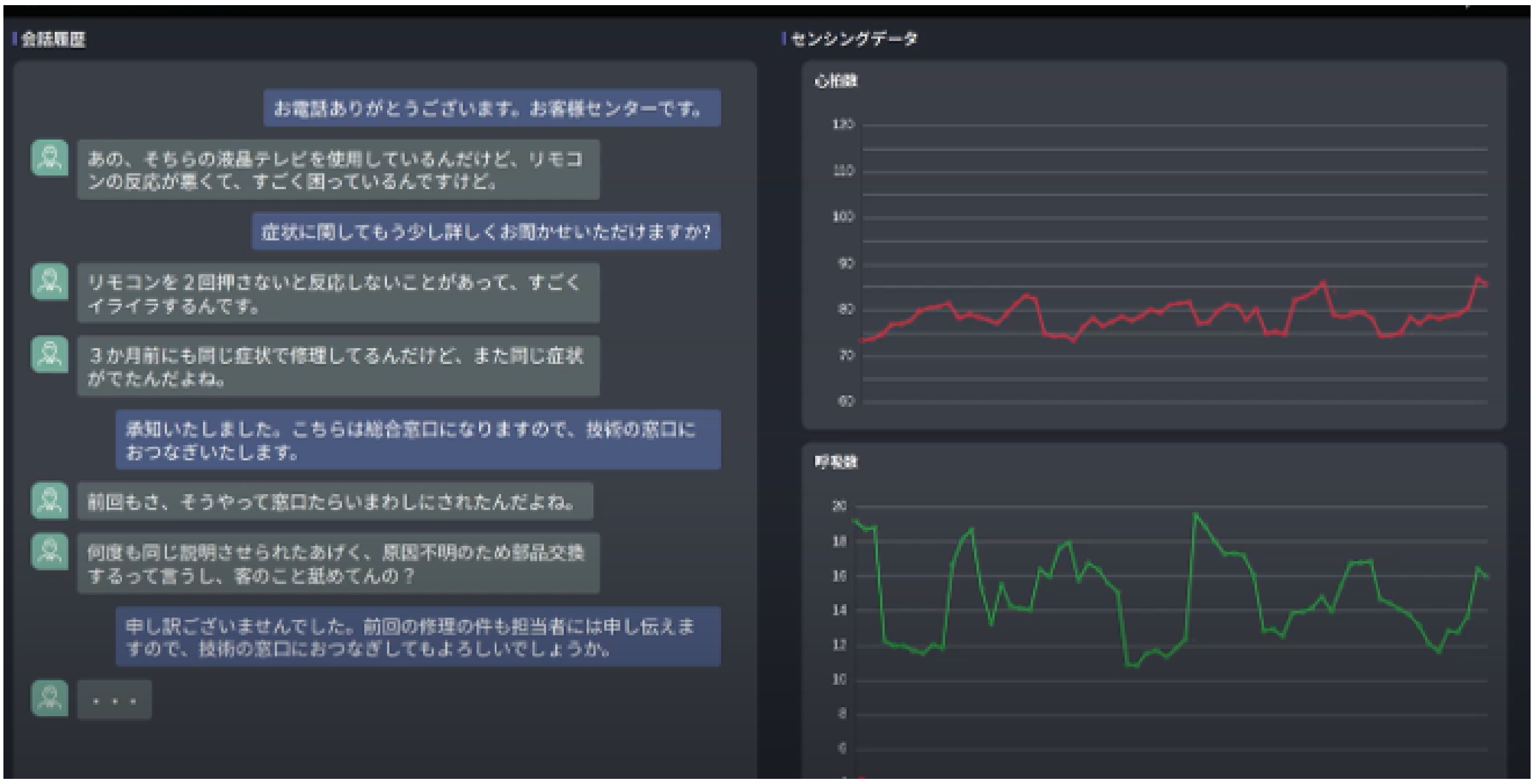

Figure S9. Original Japanese screenshot of the **Role-play phase** interface, corresponding to Figure 2 in the main text.

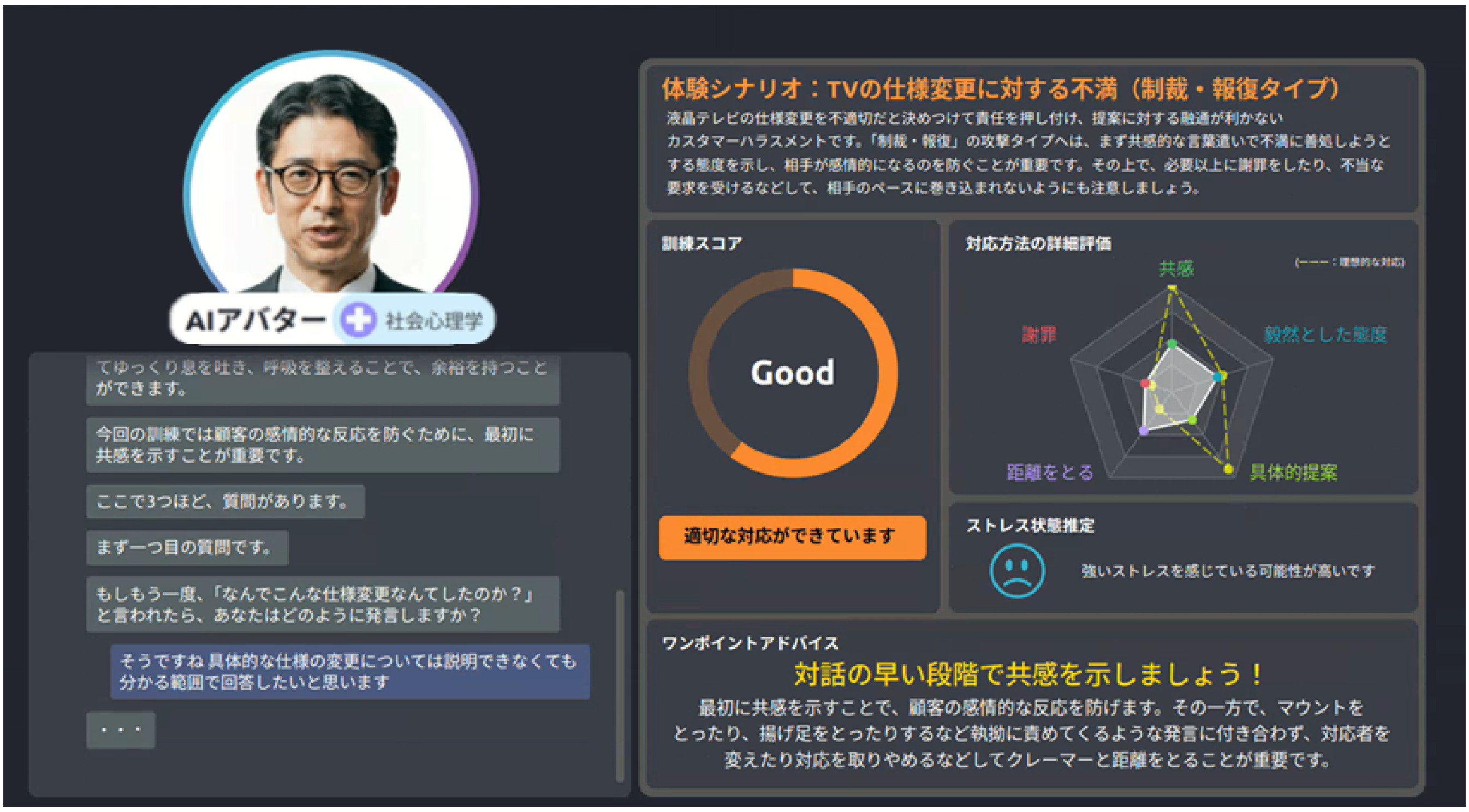

Figure S10. Original Japanese screenshot of the **Feedback phase** interface, corresponding to Figure 3 in the main text.
